# Smart network based portfolios

**DOI:** 10.1007/s10479-022-04675-7

**Published:** 2022-04-11

**Authors:** Gian Paolo Clemente, Rosanna Grassi, Asmerilda Hitaj

**Affiliations:** 1 Dipartimento di Discipline Matematiche, Finanza Matematica ed Econometria, Università Cattolica del Sacro Cuore, Milan, Italy; 2 Dipartimento di Statistica e Metodi Quantitativi, Università degli Studi di Milano-Bicocca, Milan, Italy; 3 Dipartimento di Economia, Università degli studidell’Insubria, Varese, Italy

**Keywords:** Portfolio optimization, Mean-variance, Smart Beta strategies, Networks, Dependence, Interconnectedness

## Abstract

In this article we deal with the problem of portfolio allocation by enhancing network theory tools. We propose the use of the correlation network dependence structure in constructing some well-known risk-based models in which the estimation of the correlation matrix is a building block in the portfolio optimization. We formulate and solve all these portfolio allocation problems using both the standard approach and the network-based approach. Moreover, in constructing the network-based portfolios we propose the use of three different estimators for the covariance matrix: the sample, the shrinkage toward constant correlation and the depth-based estimators . All the strategies under analysis are implemented on three high-dimensional portfolios having different characteristics. We find that the network-based portfolio consistently performs better and has lower risk compared to the corresponding standard portfolio in an out-of-sample perspective.

**Supplementary Information:**

The online version contains supplementary material available at 10.1007/s10479-022-04675-7.

## Introduction

Modern portfolio theory originates with the seminal work of Markowitz (1952). This work proposes the innovative idea of relating the return of an asset (the mean) and its risk (the variance) together with those of the other assets in the portfolio selection, through the mean-variance model. Nevertheless the prominent role in modern investment theory, this model, when applied in asset management setting, can lead to a poor out-of-sample portfolio performance, due to the estimation errors of the input parameters (see, for instance Merton 1980; Jobson and Korkie 1981). Furthermore, the risk, measured through the variance and the correlation, is based on expected values representing only a statistical statement about the future. Such measures often cannot capture the true statistical features of the risk and return which often follow highly skewed distributions.

To overcome these main drawbacks, several variations and extensions of the original methodology have been proposed in the literature. In Jagannathan and Ma (2003), a higher out-of-sample performance is derived by imposing specific constraints, and these results have been further confirmed in Behr et al. (2013) and Hitaj and Zambruno (2016). Alternative approaches deal with the problem of optimal portfolio choice by employing a Bayesian methodology to estimate unknown mean-variance parameters reducing the estimation errors. In this context, one of the most prominent is the Bayes-Stein approach based on the idea of shrinkage estimation (Jorion 1985, 1986; Bauder et al. 2018). The authors in Ledoit and Michael (2004) propose the shrinkage estimator toward the constant correlation, while in Martellini and Ziemann (2009) and Hitaj et al. (2012) this approach has been extended to higher moments such as skewness and kurtosis. Empirical analyses have shown that the use of shrinkage estimators for the mean-variance parameters often improves the out-of-sample performance (see Hitaj and Zambruno 2016, 2018). Robust optimization is widely used for dealing with uncertainty in parameters. For instance, Goldfarb and Iyengar (2003) proposed robust portfolio selection problems reformulated as second order cone programs under uncertainty structures for the market parameters. Similarly, robust mean-variance models have been put forward by Garlappi et al. (2007) and Fliege and Werner (2014). The authors in Pandolfo et al. (2020) proposed a robust estimation for mean and variance through the use of the weighted $L^p$ depth function. In particular they considered $p=2$ and performed empirical analysis using market portfolios. The authors concluded that the use of weighted $L^p$ depth function for the estimation of mean and variance is a valuable alternative in a portfolio selection problem. Exhaustive reviews about robust portfolio selection are Fabozzi et al. (2010) and Scutella and Recchia (2013) where the authors focus on the application of robust optimization only in basic mean-variance, mean-CVaR, and mean-VaR problems. Theoretical contributions can be found for other models based on either different risk measures (see Zhu and Fukushima 2009; Zymler et al. 2013; Benati and Conde 2022), or alternative performance indicators (see Kapsos et al. 2014). Concerning the empirical aspect, recently (Georgantas et al. 2021) compare the performance of several models based on well-known risk measures.

It is well known that the effects of the estimation errors of the returns are higher than the effects of the estimation errors of the covariance matrix (see, among others, Chopra and Ziemba 2013). For this reason many portfolio strategies proposed in literature have put aside returns. These are called *risk based* strategies because they rely only on the estimation of the covariance matrix. Some well-known risk based strategies are Global Minimum Variance, Equally Weighted, (DeMiguel et al. 2007), Equal Risk Contribution (Qian 2006; Maillard et al. 2010) and Maximum Diversified Portfolio (Choueifaty and Coignard 2008). The *risk based* strategies are also called *Smart Beta*^1^ strategies, as they are also proposed as alternatives to market capitalization-weighted indices, which are claimed to be not efficient (Choueifaty and Coignard 2008). The literature on constructing new portfolios able to beat a benchmark is vast and is not limited to the papers cited above. In particular, there is a wide literature on *enhanced indexing* where the objective is to outperform the index (see, e.g., Bruni et al. 2017; de Paulo et al. 2016; Dentcheva and Ruszczynski 2003; Guastaroba et al. 2016; Roman et al. 2013). We highlight that in this paper we focus only the *Smart Beta* strategies.

In the last few years the problem of asset allocation has been discussed under a different perspective. Clustering methods for financial time series has been used to build a portfolio of assets selected by the resulting partition (see Iorio et al. 2018). Another approach consists in using network theory to represent the financial market. Indeed, in network-based portfolio models, the correlation matrix is included in the network structure, in order to reproduce the dependence among the assets (see, for instance, Mantegna 1999; Onnela et al. 2003b; Pozzi et al. 2013; Zhan et al. 2015), providing in this way useful insights in the portfolio selection process.

In particular, the minimum spanning tree has been used in Onnela et al. (2003b), the authors in Pozzi et al. (2013) use Planar Maximally Filtered Graphs, while in Zhan et al. (2015) hierarchical clustering trees and neighbor-nets have been applied in order to reduce the complexity of the network, characterizing the heterogeneous spreading of risk across a financial market. The work of Peralta and Zareei (2016) establishes a bridge between Markowitz’s framework and the network theory, showing a negative relationship between optimal portfolio weights and the centrality of assets in the financial market network. As a result, the centrality measures of constructed networks can be used to facilitate the portfolio selection. A generalization to this approach has been provided in Vỳrost et al. (2019).

Recently, an alternative methodology to tackle the asset allocation problem using the network theory has been proposed in Clemente et al. (2019). Specifically, the authors catch how much a node is embedded in the system, by adapting to this context the clustering coefficient, a specific network index (see Barrat et al. 2004; Clemente and Grassi 2018; Fagiolo 2007; McAssey and Bijma 2015; Wasserman and Faust 1994; Watts and Strogatz 1998), meaningful in financial literature to assess systemic risk (Bongini et al. 2018; Minoiu and Reyes 2013; Tabak et al. 2014). The underlying structure of the financial market network is used as an effective tool in enhancing the portfolio selection process. In particular, the optimal allocation is obtained by maximizing a specific objective function that takes into account the interconnectedness of the system, unlike the classical global minimum variance model that is based only on the pairwise correlation between assets. Furthermore, in constructing the dependence structure of the portfolio network, various dependence measures are tested, namely, the Pearson correlation, Kendall correlation and lower tail dependence. All these measures are estimated using the sample approach. The results obtained in Clemente et al. (2019) show that, independently from the length of the rolling window and from the used dependence structure, the network-based portfolio leads to better out-of-sample performance compared with the classical approach.

The aim of this paper is to move one step further by enhancing the role of the network theory in solving portfolio allocation problem. The main contribution is the extension of the existing network-based approaches to different portfolio selection problems where the objective function depends on the variance-covariance matrix. In particular, we contribute to the existing literature along various dimensions.

On the one hand, we exploit the network theory constructing the Smart Beta strategies and the mean-variance portfolio, where alternative values of the trade-off parameter are considered. On the other hand, we extend the network-theory to the estimation of the variance-covariance matrix using alternative methodologies, as the shrinkage (toward the constant correlation) and the weighted $L^p$ depth function.

The out-of-sample performance of the proposed methodology is empirically tested. Specifically, the Pearson correlation is used in order to capture the dependence structure of the portfolio network. Moreover, we apply the network theory to various well-known models in which the estimation of the correlation matrix is a building block in the portfolio optimization. We consider in this paper Equally Risk Contribution, Maximum Diversified Portfolio, Global Minimum Variance, and the mean-variance model. In this last case we consider different levels of the trade-off parameter. Moreover, since recent academic papers and practitioner publications suggest that equal-weighted portfolios appear to outperform various other price-weighted or value-weighted strategies (see, e.g., DeMiguel et al. 2007), we also include the Equally Weighted (EW) portfolio in our analysis.

Through empirical analyses, we test the impact of the estimation method on both the *standard* and *network-based* portfolios. For the sake of completeness, three different high-dimensional datasets with different characteristics are considered. The first dataset is composed by 266 among largest banks and insurance companies in the world, whose daily returns have been collected in the time-period ranging from January 2001 to December 2017. The second dataset is composed by the components of the *S &P 100* index (with Bloomberg ticker OEX) and the third dataset includes the components of the *Nikkei-225 Stock Average* (with Bloomberg ticker NKY) . The *OEX* and the *NKY* datasets contain daily returns in the time-period ranging from January 2001 to July 2021. All the obtained portfolios are compared in an out-of-sample perspective using some well-known performance measures. Main results show that, in the majority of the cases, the use of network-based approach leads to higher out-of-sample performances and lower volatility with respect to the corresponding *sample* strategy. The network-based portfolio is more robust with respect to the standard approach being only slightly affected by the estimation method of the covariance matrix. The out of-sample results suggest that the network-based strategy represents a viable alternative to classical portfolio strategies.

The remainder of the paper is organized as follows. Sect. 2 briefly recalls the investor’s problems for each strategy under analysis. Section 2 explains the two estimation methods used for the covariance matrix. Section 3 explains in detail the approach of portfolio selection via network theory. Section 4 presents the empirical analysis and Sect. 5 draws main conclusions.

## Portfolio selection strategies

In this section, we briefly set out the strategies used in the rest of the paper for the empirical analysis. We first introduce what we refer to as standard strategies. We start with the mean-variance problem and then we describe the most important Smart Beta approaches proposed as alternatives to the market capitalization-weighted indices, in the equity world.

### Mean-variance (MV)

Let us first introduce the standard *mean-variance* model for a portfolio with *N* risky assets. Let $R_i$ be the random variable (r.v.) of daily log returns. Let ${{\textbf {r}}}=[r_{i}]_{i=1,\ldots ,N}$ be the returns’ vector observed in a specific time period/window (*w*) and ${\varvec{\mu }}$ (${\varvec{\Sigma }}$) be the mean vector (variance-covariance matrix) between assets estimated in the same period. Let ${\mathbf {e}}$ and ${\mathbf {x}}=[x_i]_{i=1,\ldots ,N}$ be, respectively, the vector of ones and the vector of portfolio weights, i.e. the proportional investments in the *N* risky assets. We denote with $\mu _p={{\textbf {x}}}^{T} {\varvec{\mu }}$, $\sigma _i$ and $\sigma _P={\sqrt{{\mathbf {x}}^{T}{\varvec{\Sigma }}\ {\mathbf {x}}}}$ the portfolio mean, the standard deviation of the $i^{th}$ asset and the standard deviation of the portfolio, respectively. We recall that all optimization models considered in this paper include realistic investment constraints such as budged constraint (i.e. ${\mathbf {e}}^T{\mathbf {x}}=1$) and non-short selling constraints $x_i \ge 0$ $\forall i=1,\ldots ,N$ since many institutional investors are restricted to long positions only.

The mean-variance model proposed in Markowitz (1952) consists in optimizing a trade-off between risk and return. The standard mean-variance (MV) portfolio optimization problem is given by:

1 $$\begin{aligned} \left\{ \begin{array}{ll} \min \limits _{{\mathbf {x}}} \ \ \ \lambda {{\textbf {x}}}^{T}{\varvec{\Sigma }}{{\textbf {x}}} - (1-\lambda ){{\textbf {x}}}^{T} {\varvec{\mu }} \\ {\mathbf {e}}^T{\mathbf {x}}=1\\ 0 \le x_i \le 1, &{}\quad {i=1}, \ldots , {N} \end{array}, \right. \end{aligned}$$

where $\lambda \in [0,\ 1]$ expresses the trade-off between risk and return of the portfolio. It is possible to compute alternative points on the efficient frontier by solving problem (1) for different levels of $\lambda$. The level of $\lambda$ plays a crucial role in the portfolio diversification and it can be set by the decision manager according to its preferences. A risk-prone investor may choose a low level of $\lambda$. The extreme case $\lambda =0$ will lead to a highly concentrated portfolio as the investor is ignoring the risk and the portfolio will be concentrated to the asset with the higher mean. On the contrary, for $\lambda =1$ the portfolio will be diversified as in this case the manager is ignoring the portfolio return and seeking the portfolio with the lower risk. Therefore, the lower is $\lambda$ the less diversified may be the portfolio. For this reason in the empirical analysis we considered four alternative levels, $\lambda \in \left\{ 0.2\ \ 0.4 \ \ 0.6 \ \ 0.8 \right\}$

It is evident from (1) that the MV portfolio optimization relies on estimators of the means and covariances of the asset returns. This means that the MV portfolio strongly depends on the input data, see among others Jorion (1992) and Chopra and Ziemba (2013). The estimation methods used in this paper will be explained in more details in Sect. 2.1

#### Global minimum-variance portfolio (GMV)

The GMV strategy selects weights that minimize the variance of the portfolio ignoring completely the portfolio return. The GMV optimization problem is formulated as:

2 $$\begin{aligned} \left\{ \begin{array}{ll} \min \limits _{{\mathbf {x}}} \ \ \ {{\textbf {x}}}^{T}{\varvec{\Sigma }}{{\textbf {x}}}\\ {\mathbf {e}}^T{\mathbf {x}}=1\\ 0 \le x_i \le 1, &{} {i=1, \dots ,N} \end{array}, \right. \end{aligned}$$

#### Equally weighted portfolio (EW)

The EW strategy consists in holding a portfolio characterized by the same weight $\frac{1}{N}$ in each component. In the literature, it has been empirically showed that the EW portfolios perform better than many other quantitative models, with higher Sharpe Ratio and Certainty Equivalent return (see DeMiguel et al. 2007). Being the weights equally allocated among the assets, this strategy disregards the data and, of course, it does not require any optimization or estimation procedure.

#### Equal risk contribution portfolio (ERC)

The equal risk contribution strategy (ERC) is characterized by weights such that each asset provides the same contribution to the risk of the portfolio. The marginal contribution of the asset *i* to the portfolio risk is:

$$\begin{aligned} \partial _{x_{i}}\sigma _{P}=\frac{\partial \sigma _{P}}{\partial x_{i}}=\frac{\left( {\varvec{\Sigma }}\ {{\textbf {x}}}\right) _{i}}{\sqrt{{\mathbf {x}}^{T}{\varvec{\Sigma }}\ {\mathbf {x}}}}. \end{aligned}$$

Hence, $\sigma _{i}(x)= x_{i} \partial _{x_{i}}\sigma _{P}$ represents the risk contribution of the $i^{th}$ asset to the portfolio *P*. The authors in Maillard et al. (2010) proved that the portfolio risk can be expressed as:

$$\begin{aligned} \sigma _{P}=\sum _{i}^{N} \sigma _{i} (x) \end{aligned}$$

that is the sum of risk contributions of the assets. The characterizing property of the ERC strategy is that weights are such that $\sigma _{i} (x)=\sigma _{j}(x)$ $\forall \ i,j$. The result is a portfolio extremely diversified in terms of risk.

To obtain the optimal weights we have to solve an optimization problem consisting in minimizing the sum of all squared deviations under budged and non short-selling constraints. The mathematical formulation is the following:

3 $$\begin{aligned} \left\{ \begin{array}{l} \underset{{{\textbf {x}}}}{\min }\ \sum _{i=1}^N \ \sum _{j=1}^N \ \left( x_{i}({\varvec{\Sigma }}\ {{\textbf {x}}})_{i}-x_{j}({\varvec{\Sigma }}\ {{\textbf {x}}})_{j}\right) ^2\\ {\mathbf {e}}^T{\mathbf {x}}=1 \\ x_i \ge 0 \ \ i=1,...N \end{array} \right. , \end{aligned}$$

#### Maximum diversified portfolio (MDP)

The Maximum Diversification approach aims to construct a portfolio that maximizes the benefits from diversification. This goal can be achieved by solving a maximization problem where the objective function is given by the so-called Diversification Ratio $DR=\frac{\sum _{i=1}^N{x_{i}\sigma _{i}}}{\sigma _P}$, under the usual constraints. The mathematical formulation for the MDP strategy is:

4 $$\begin{aligned} \left\{ \begin{array}{l} \underset{{\mathbf {x}}}{\max }\ \frac{\sum _{i=1}^N{x_{i}\sigma _{i}}}{\sqrt{{\mathbf {x}}^{T} \Sigma {\mathbf {x}}}}\\ {\mathbf {e}}^T{\mathbf {x}}=1 \\ x_i \ge 0 \ \ i=1,...N \end{array} \right. \end{aligned}$$

This approach creates portfolios characterized by minimally correlated assets, providing lower risk levels and higher returns than market cap-weighted portfolios strategies (see Choueifaty and Coignard 2008).

### Estimation methods for covariance matrix

Unlike the investment problems (2), (3) and (4), in which only the estimate of the covariance matrix between assets in a given time interval is needed, we have to estimate both the covariance matrix and the mean vector in order to solve problem (1). A common way to estimate them is through the *sample approach*. This method allows to obtain each component ${\hat{\mu }}_i$ and ${\hat{\sigma }}_{i,j}$ by means of classical unbiased estimators. However, it is well known that the sample estimator of historical returns is likely to generate high sampling error. For this reason several methods have been introduced in order to improve the estimation of moments and comoments (see, among others, (Jorion 1985, 1986; Ledoit and Michael 2004; Martellini and Ziemann 2009) and Pandolfo et al. 2020). These methods are grounded on the idea of imposing some structure on the moments (comoments) with the aim to reduce the number of parameters, leading in this way to a reduction of the sampling error at the cost of specification error.

It is worth stressing that in this work we mainly focus on the estimation of the covariance matrix. For this reason, we estimate the mean only through the sample approach, whereas we pay more attention to the estimate of the covariance matrix, considering also the shrinkage toward the constant correlation (CC) method (see Elton and Gruber 1973; Ledoit and Michael 2004) and *wighted* $L^P$ *data depth function* (see Pandolfo et al. 2020). The idea of the CC approach is to estimate the covariance based on the fact that the correlation is assumed constant for each pair of assets, and it is given by the average of all the sample correlation coefficients (see Elton and Gruber 1973). The covariance between two assets is then computed as

5 $$\begin{aligned} \sigma _{i,j}^{CC}={\hat{\sigma }}_{i}{\hat{\sigma }}_{j}\frac{1}{N(N-1)} \sum _{\begin{array}{c} {i,j=1}\\ i \ne j \end{array}}^N{\left( {\hat{\rho }}_{i,j}\right) }, \end{aligned}$$

where ${\hat{\rho }}_{i,j}$ is the sample correlation between assets *i* and *j*.

This approach resizes the problem, as only one correlation coefficient and *N* standard deviations have to be estimated. The ${\varvec{\Sigma ^{CC}}}$ covariance matrix, constructed by using previous formula, is characterized by a lower estimation risk due to the assumed structure, nevertheless it involves some misspecification in the artificial structure imposed by this estimator. In the attempt to find a trade-off between the sample risk and the model risk, the authors in Ledoit and Michael (2004) introduce the asymptotically optimal linear combination of the sample estimator and the structured estimator (in our case, the CC estimator) in the context of the covariance matrix, with the weight given by the optimal shrinkage intensity $\kappa$.^2^ Therefore, the shrinkage toward CC covariance matrix is given by:

6 $$\begin{aligned} \sigma _{i,j}^{shrink}=\kappa \sigma _{i,j}^{CC}+ (1-\kappa ) {\hat{\sigma }}_{i,j}. \end{aligned}$$

Recently, in Pandolfo et al. (2020) the robust estimation of the mean and variance based on statistical data depth functions has been used in finance. Let ${\mathcal {F}}$ be the class of distributions on the Borel sets of ${\mathbb {R}}^N$ ($N > 1$) and $F_{{\mathbf {R}}}$ be the joint distribution of a random vector ${\mathbf {R}} \in {\mathbb {R}}^N$

A statistical data depth function is a bounded and non-negative function $D(\cdot ;\cdot \ ): {\mathbb {R}}^N\times {\mathcal {F}} \rightarrow {\mathbb {R}}$ which satisfies the following desirable properties:

- *Affine Invariance* $D({\mathbf {A}}{\mathbf {r}}+{\mathbf {b}};\ F_{{\mathbf {A}}{\mathbf {R}}+{\mathbf {b}}})=D({\mathbf {r}},F_{{\mathbf {R}}})$ holds for any vector ${\mathbf {R}} \in {\mathbb {R}}^N$, for any nonsingular $N\times N$ matrix ${\mathbf {A}}$ and for any vector ${\mathbf {b}}, {\mathbf {r}} \in {\mathbb {R}}^N$. This implies that the depth of ${\mathbf {r}}$ should be invariant to scale and location.
- *Maximality at Center* Let ${\mathbf {r}}^{\star }\in {\mathbb {R}}^N$ be a uniquely defined *center* (e.g., the point of symmetry with respect to some notion of symmetry) of $F_{{\mathbf {R}}}$ then $D({\mathbf {r}}^{\star };\ F_{{\mathbf {R}}})=\underset{{\mathbf {r}}\in {\mathbb {R}}^N}{\sup }\ {D({\mathbf {r}};\ F_{{\mathbf {R}}})}$; this means that *D* attains the maximum at the center.
- *Monotonicity Relative to Deepest Point* $D({\mathbf {r}};\ F_{{\mathbf {R}}})\le D({\mathbf {r}}^{\star }+\beta ({\mathbf {r}}-{\mathbf {r}}^{\star });\ F_{{\mathbf {R}}})$ for any $\beta \in [0,\ 1]$ and ${\mathbf {r}} \in {\mathbb {R}}^N$. This implies that as the ${\mathbf {r}}$ moves away from the center (${\mathbf {r}}^{\star }$) the depth at ${\mathbf {r}}$ should decrease monotonically.
- *Vanishing at Infinity* $D({\mathbf {r}};\ F_{\mathbf {R}}) \rightarrow 0$ as $||{\mathbf {r}}|| \rightarrow \infty$. The depth of ${\mathbf {r}}$ should approaches 0 as $||{\mathbf {r}}||$ approaches infinity.

As in Pandolfo et al. (2020), in the empirical part of this paper we use a statistical data depth function based on the distance approach, where the non negative distance function belongs to the $L^p$ norm $(p>0)$.

A distance function of ${\mathbf {r}}\in {\mathbb {R}}^N$ from ${\mathbf {R}}$, based on the $L^p$ norm, can be written as:

7 $$\begin{aligned} {D_{L^p}({\mathbf {r}}, F_{\mathbf {R}})=\frac{1}{1+{\mathbb {E}}\left| \left| {\mathbf {r}}-{\mathbf {R}}\right| \right| _p }.} \end{aligned}$$

We recall that the distance $D_{L^p}$ does not possess the affine invariance property. As reported in Zuo (2004) and Zuo et al. (2004), different distances with respect to the data may not have the same importance. For this reason in order to obtain location and scatter estimators, designed to achieve greater robustness, the authors proposed a weighted $L^p$ depth function, given by:

8 $$\begin{aligned} {WD_{L^p}({\mathbf {r}}, F_{\mathbf {R}})=\frac{1}{1+{\mathbb {E}}\left[ W\left( \left| \left| {\mathbf {r}}-{\mathbf {R}}\right| \right| _p \right) \right] },} \end{aligned}$$

where $W(\cdot )$ is a weight function, non-decreasing and continuous on $[0, \ \infty )$, that down-weights outlying observations.

Let $({\mathbf {r}}_1, \ldots , {\mathbf {r}}_d)'$ be a *N*-variate simple random sample of size *d* from ${\mathbf {R}}\sim F_{\mathbf {R}}$. The authors in Pandolfo et al. (2020) used the weighted $L^2$ data depth function to obtain robust estimates of the mean ($\mathbf {\mu }^{WD_{L^2}}$) and covariance matrix (${\Sigma }^{WD_{L^2}}$) of the asset returns, given respectively by:

9 $$\begin{aligned} {\mathbf {\mu }^{WD_{L^2}}=\frac{\sum _{t=1}^{d}W_1\left( D({\mathbf {r}}_t, F_{{\mathbf {R}}}) \right) {\mathbf {r}}_t }{\sum _{t=1}^{d}W_1\left( D({\mathbf {r}}_t, F_{{\mathbf {R}}}) \right) }} \end{aligned}$$

and

10 $$\begin{aligned} {{\Sigma }^{WD_{L^2}}=\frac{\sum _{t=1}^{d}W_2\left( D({\mathbf {r}}_t, F_{{\mathbf {R}}}) \right) \left( {\mathbf {r}}_t-\mathbf {\mu }^{WD_{L^2}}\right) \left( {\mathbf {r}}_t-\mathbf {\mu }^{WD_{L^2}}\right) ' }{\sum _{t=1}^{d}W_2\left( D({\mathbf {r}}_t, F_{{\mathbf {R}}}) \right) }} \end{aligned}$$

where $W_1\left( D({\mathbf {r}}_t, F_{{\mathbf {R}}})\right)$ and $W_2\left( D({\mathbf {r}}_t, F_{{\mathbf {R}}})\right)$ are two weighted functions non decreasing and continuous on $\left[ 0, \ \infty \right]$. $W_j\left( D(\cdot , \cdot )\right) , \ \ \text {for} \ \ j=1,2,$ is given in Pandolfo et al. (2020) by:

11 $$\begin{aligned} W_j\left( h\right) =\left\{ \begin{array}{l} \frac{\exp \left( -k\left( 1-(\frac{h}{c})^{2j} \right) ^{2j} \right) -\exp {(-k)}}{1-exp{(-k)}} \ \ \ \ \text {if}\ \ \ \ h<c \\ \ \ \ \ 1 \ \ \ \ \ \ \ \ \ \ \ \ \ \ \ \ \ \ \ \ \ \ \ \ \ \ \text {if}\ \ \ h \ge c \end{array}, \right. \end{aligned}$$

where *c* is the median of the data depth function and the value *k* determines how heavily the weight function penalizes as *h* get away from *c*. Following (Pandolfo et al. 2020), in the empirical part we set $p=2$ and $k=3$.

The advantages of the robust approaches considered in this paper are that both are non-parametric approaches and are less sensitive to changes in the asset return distribution compared to the sample estimate.

We highlight that in the empirical part the depth data function is $D({\mathbf {r}};\ {\hat{F}}_{{\mathbf {R}},d})$, where ${\hat{F}}_{{\mathbf {R}},d}$ is the empirical joint distribution of ${\mathbf {R}}$ estimated from the observed returns $({\mathbf {r}}_1, \ldots , {\mathbf {r}}_d)'$.

In the following section we recall the network correlation-based portfolio model and explain how the three estimators of the variance-covariance matrix (sample, shrinkage toward CC and $WD_{L^2}$) can be used.

## Optimal portfolio via network theory

The portfolio selection problem and its variants can be formulated in a networks context and several researchers dealt with the assets allocation problem using network theory tools, contributing to the related literature (Peralta and Zareei 2016; Clemente et al. 2019; Li et al. 2019; Pozzi et al. 2013). All these articles share the same framework, namely the financial market is represented as a network, in which nodes are assets and weights on the edges identify a dependence measure between returns.

We describe in this section the approach proposed by Clemente et al. (2019). The authors formulate an investment strategy that benefits from the knowledge of the dependency structure that characterizes the market. Unlike the risk-based strategies, based on an objective function that accounts for pairwise correlations among assets, the objective function considers here the interconnectedness of the whole system.

In order to make the paper self-consistent, we briefly remind some preliminary definitions and notations about networks. A network $G=(V,E)$ consists in a set *V* of nodes and a set *E* of edges between nodes, where the edge (*i*, *j*) connects a pair of nodes *i* and *j*. If $(j,i)\in E$ whenever $(i,j)\in E$, the network is undirected. A network is complete if every pair of vertices is connected by an edge. We denote with ${\mathbf {A}}$ the real *N*-square matrix whose elements are $a_{ij}=1$ whenever $(i,j)\in E$ and 0 otherwise (the adjacency matrix). A network is weighted if a weight $w_{ij} \in {\mathbb {R}}$ is associated to each edge (*i*, *j*). In this case, both adjacency relationships between vertices of *G* and weights on the edges are described by a non negative, real *N*-square matrix ${{\textbf {W}}}$ (the weighted adjacency matrix). We denote with $k_{i}$ and $s_{i}$ the degree and strength of the node *i* $(i=1,...,N)$, respectively.

Relationships between assets are quantified through three different levels of dependence. For the sake of brevity, we report here only the approach referring to the classical linear correlation network, which is the most used dependence measure in the literature. Since all assets are correlated in the market, the correlation structure is represented through a weighted, complete and undirected network *G*, where weights on the edges are given by the Pearson correlation coefficient between them, that is $w_{ij}=\rho \left( R_{i},R_{j}\right)$ $\forall i \ne j$. In order to assure nonnegative weights, a distance can be associated with the correlation coefficient (see Giudici and Spelta 2016; Mantegna 1999; Onnela et al. 2003a). In our case, this transformation does not affect the results in terms of optimal portfolio.

The extension of the pairwise correlations, included in the quadratic form of the problem (2), to a general intercorrelation among all stocks at the same time is obtained optimizing a function that includes the clustering coefficient. The classic clustering coefficient and its variants defined in the literature (see Watts and Strogatz 1998; Cerqueti et al. 2018; Fagiolo 2007; Clemente and Grassi 2018) are not computable for complete networks, then we have to adapt its formulation to this framework.

Following a similar procedure to that proposed by McAssey and Bijma (2015), a threshold $s\in [-1,1]$ is introduced on the matrix ${\mathbf {W}}$ in order to define the new matrix ${\mathbf {A}}_{s}$, whose elements $a_{ij}^s$ are

12 $$\begin{aligned} a_{ij}^s = {\left\{ \begin{array}{ll} 1 &{} \text{ if } w_{ij} \ge s \\ 0 &{} \text{ otherwise } \end{array}\right. }. \end{aligned}$$

${\mathbf {A}}_s$ is the adjacency matrix describing the existing links in the network with weights $w_{ij}$ at, or above the threshold *s*. Through this matrix we are selecting the strongest edges, namely those greater than a given threshold, bringing out the mean cluster prevalence of the network. On this new network we compute the clustering coefficient proposed in Watts and Strogatz (1998) and then we repeat the process, varying the threshold *s*. The clustering coefficient $C_{i}$ for a node *i* corresponding to the graph is the average of $C_{i}({\mathbf {A}}_{s})$ overall $s\in [-1,1]$:

13 $$\begin{aligned} C_{i}=\int _{-1}^{1}C_{i}({\mathbf {A}}_{s})ds \end{aligned}$$

Since $0\le C_i\le 1$, $C_{i}$ is well-defined. Now, we define the *N*-square matrix ${\mathbf {C}}$, of entries

14 $$\begin{aligned} c_{ij} = {\left\{ \begin{array}{ll} C_{i}C_{j} &{} \text{ if } i \ne j \\ 1 &{} \text{ otherwise } \end{array}\right. }. \end{aligned}$$

This matrix accounts for the level of interconnection for all pairs with the whole system, therefore, it can be used to construct the matrix

15 $$\begin{aligned} {\mathbf {H}}={\varvec{\Delta }}^T{\mathbf {C}}{\varvec{\Delta }} \end{aligned}$$

where ${\varvec{\Delta }}=diag(s_{i})$ is a diagonal matrix with diagonal entries

$$\begin{aligned} s_i=\frac{{\hat{\sigma }}_{i}}{\sqrt{\sum _{i=1}^{N}{\hat{\sigma }}_{i}^{2}}}. \end{aligned}$$

Notice that the element $s_{i}$ considers the contribute of the standard deviation of the return *i* to the total standard deviation, computed in case of independence. In Clemente et al. (2019), the authors solve the optimization problem defined in (2) replacing the covariance matrix ${\varvec{\Sigma }}$ with ${\mathbf {H}}$.

The main difference between the classical and the network portfolio selection problem is due to the use of the interconnectedness matrix in order to consider how much each couple of assets is related to the system. In particular, being ${\mathbf {C}}$ dependent on a network-based measure of systemic risk (i.e. the clustering coefficient), we are implicitly including a measure of the state of stress of the financial system in the time period.

## Dataset description and empirical analysis

### Dataset description

The goal of this section is to examine the out-of-sample properties of the Smart Beta and mean-variance network-based portfolios in which the covariance matrix is estimated using the network theory though the methodology described in Sect. 3. In particular, we make a comparison between network-based portfolios and standard portfolio strategies, where the three previously described estimators of the covariance matrix are considered: the sample, the shrinkage and the weighted $L^2$ depth function. We summarize in Table 1 the alternative asset allocation models applied in this analysis.

**Table 1:** List of asset allocation models considered in the empirical study

Number	Model	Label
1	Standard sample based mean-variance (MV)	S-sample MV
2	Standard shrinkage toward constant correlation based mean-variance (MV)	S-shrinkage MV
3	Standard $WD_{L^2}$ based mean-variance (MV)	S-$WD_{L^2}$ MV
4	Network sample based mean-variance (MV)	NB-sample MV
5	Network shrinkage toward constant correlation based mean-variance (MV)	NB-shrinkage MV
6	Network $WD_{L^2}$ based mean-variance (MV)	NB-$WD_{L^2}$ MV
7	Standard sample based Maximum Diversified Portfolio (MDP)	S-sample MDP
8	Standard shrinkage toward constant correlation based MDP	S-shrinkage MDP
9	Standard $WD_{L^2}$ based Maximum Diversified Portfolio (MDP)	S-$WD_{L^2}$ MDP
10	Network sample based MDP	NB-sample MDP
11	Network shrinkage toward constant correlation based MDP	NB-shrinkage MDP
12	Network $WD_{L^2}$ based Maximum Diversified Portfolio (MDP)	NB-$WD_{L^2}$ MDP
13	Standard sample based Equally Risk Contribution (ERC)	S-sample ERC
14	Standard shrinkage toward constant correlation based ERC	S-shrinkage ERC
15	Standard $WD_{L^2}$ based Equally Risk Contribution (ERC)	S-$WD_{L^2}$ ERC
16	Network sample based ERC	NB-sample ERC
17	Network shrinkage toward constant correlation based ERC	NB-shrinkage ERC
18	Network $WD_{L^2}$ based Equally Risk Contribution (ERC)	NB-$WD_{L^2}$ ERC
19	Standard sample based Global Minimum Variance (GMV)	S-sample GMV
20	Standard shrinkage toward constant correlation based GMV	S-shrinkage GMV
21	Standard $WD_{L^2}$ based Global Minimum Variance (GMV)	S-$WD_{L^2}$ GMV
22	Network sample based GMV	NB-sample GMV
23	Network shrinkage toward constant correlation based GMV	NB-shrinkage GMV
24	Network $WD_{L^2}$ based Global Minimum Variance (GMV)	NB-$WD_{L^2}$ GMV
25	Equally Weighted	EW

The last column of the table indicates the label used to refer to each strategy in the empirical section, where the performance of the various approaches is compared

As a robustness check we consider three large-dimensional portfolios with different characteristics. The investment universe of the first portfolio is composed by 266 among largest banks and insurance companies in the world.^3^ In particular we have 120 insurers and 144 banks. The dataset of this portfolio contains daily returns in the time-period ranging from January 2001 to December 2017. The investment universe of the second portfolio consists of the components of the S &P 100 index. A third portfolio consists of the components of *Nikkei-225 Stock Average*, that considers the 225 stocks selected from domestic common stocks in the first section of the Tokyo Stock Exchange. Last two portfolios contain daily returns in the time-period ranging from January 2001 to August 2021.^4^

All the portfolios discussed in this paper are analysed and compared in an out-of-sample perspective. In particular the first four moments, the Sharpe Ratio (SR), the Omega Ratio (OR) the Information Ratio (IR) and the out-of-sample performance are used to compare the portfolios. All these aspects are investigated through a rolling window methodology, which is characterized by an in-sample period of length *d* and an out-of-sample period of length *k*.^5^ This means that the first in-sample window of width *d* contains the observations of all the components in the portfolio from $t=1$ to $t=d$. The dataset of the first in-sample window is used to estimate the optimal weights, using the different portfolio selection models considered in this paper and listed in Table 1. These optimal weights are then invested in the out-of-sample period, from $t=d+1$ to $t=d+k$, where the out-of-sample performance is computed. The process is repeated rolling the window *k* steps forward. Hence, weights are updated by solving the optimal allocation problem in the new subsample and the performance is estimated once again using data from $t=d+k+1$ to $d+2k$. Repeating these steps until the end of the dataset is reached, we buy-and-hold the portfolios and we record out-of-sample portfolio returns in each rebalancing period.

To ensure the robustness of our results, we analyse three alternative estimation windows; namely, 6 months in-sample and 1 month out-of-sample, 1 year in-sample and 1 month out-of-sample and two years in-sample and 1 month out-of-sample.

### Performance measures

In order to assess the magnitude of potential gains/losses that can be attained by an investor adopting a network-based portfolio selection, we implement an out-of-sample analysis. For this reason several performance measures are calculated. First, for each optimization strategy, we compute the first four moments of the out-of-sample portfolio returns. Further, for each strategy *j*, we determine the out-of-sample Sharpe Ratio of the optimal portfolio^6^:

$$\begin{aligned} SR_j^{\star }=\frac{\mu _{p_j} ^{\star }-\mu _f}{\sigma _{p_j}^{\star }}, \end{aligned}$$

where $\mu _{p_j}^{\star }$ and $\sigma _{p_j}^{\star }$ are, respectively, the average return and the standard deviation of the optimal portfolio according to the strategy *j* and $\mu _f$ indicates the average risk-free rate.^7^ This ratio measures the average return of a portfolio in excess of the risk-free rate, also called the risk premium, as a fraction of the portfolio total risk, measured by its standard deviation. As alternatives performance measures we also calculate the Information ratio (IR) and the Omega ratio (OR). The Information ratio of the optimal portfolio is defined as:

$$\begin{aligned} IR_j^{\star }=\frac{\mu _{p_j} ^{\star }-\mu _{p_{ref}} ^{\star }}{\sigma (r_{p,j}^{\star }-r_{p_{ref}}^{\star })}. \end{aligned}$$

where $\mu _{p_{ref}} ^{\star }$ is the average return of the reference portfolio and $r_{p,j}^{\star },r_{p_{ref}}^{\star }$ represent the out-of-sample time series of optimal portfolio returns corresponding to a strategy *j* and the reference strategy, respectively.

Once identified the reference portfolio, managers seek to maximize $IR_{j}$, i.e. to reconcile a high residual return and a low tracking error. This ratio allows to check if the risk taken by the manager in deviating from the reference portfolio is sufficiently rewarded.

The *Omega Ratio* has been introduced by Keating and Shadwick in Keating and Shadwick (2002) and it is defined as:

$$\begin{aligned} OR_j^{\star } = \frac{\int _{\epsilon }^{+\infty } (1-F_j(x))\,dx}{\int _{-\infty }^{\epsilon } F_j(x)dx}=\frac{{\mathbb {E}}\left( r^{\star }_{p_j}-\epsilon \right) ^+}{{\mathbb {E}}\left( \epsilon -r^{\star }_{p_j}\right) ^+}, \end{aligned}$$

where $F_j(x)$ is the cumulative distribution function of the portfolio returns for a strategy *j* and $\epsilon$ is a specified threshold.^8^ Returns below (respectively above) the threshold are considered as losses (respectively gains). In general, a value of the $OR_{j}$ greater than 1 indicates that strategy *j* provides more expected gains than expected losses. The portfolio with the highest ratio will be preferred by the investor. The $OR_{j}$ implicitly embodies all the moments of the return distribution without any a-priori assumption.

### Empirical results

As previously explained, for robustness purposes we consider three large-dimensional datasets. The first is the Banks and Insurers dataset composed by 266 among largest banks and insurance companies in the world. The second is composed by the assets of the S &P 100 index and the third regards the constituents of the Nikkei-225 Stock Average (NKY). This empirical analysis is based on a buy and hold strategy. For the sake of completeness we consider three alternative strategies, with an in-sample period of two years, one year and six months, respectively, and an out-of-sample period of one month. For the sake of brevity, we report in the following the results obtained for the NKY dataset with a rolling window of one year in-sample and one month out-of-sample. However, all the detailed statistics of the three analysed portfolios, for all strategies and estimation methods used for the covariance matrix are reported in the Supplementary Material.

**Fig. 1:**
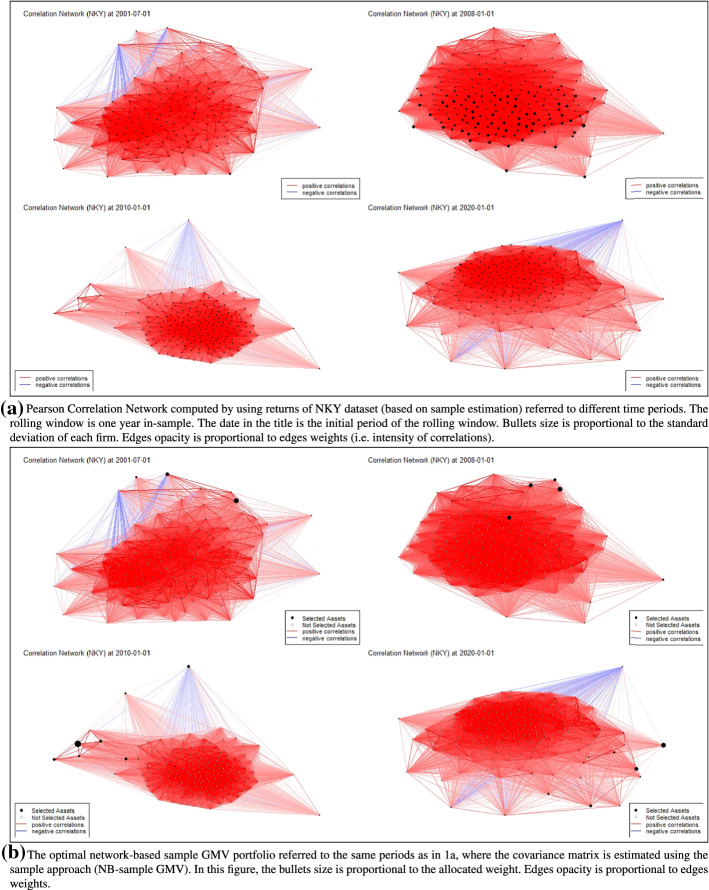
**a** Pearson Correlation Network computed by using returns of NKY dataset (based on sample estimation) referred to different time periods. The rolling window is one year in-sample. The date in the title is the initial period of the rolling window. Bullets size is proportional to the standard deviation of each firm. Edges opacity is proportional to edges weights (i.e. intensity of correlations). **b** The optimal network-based sample GMV portfolio referred to the same periods as in **a**, where the covariance matrix is estimated using the sample approach (NB-sample GMV). In this figure, the bullets size is proportional to the allocated weight. Edges opacity is proportional to edges weights

The proposed network approaches allow to visualize the portfolio composition and the dependence structure between assets. To have a preliminary idea of the results, we depict in Fig. 1a the structure of the network of the NKY dataset based on the correlation matrix, obtained via sample estimation, at different time periods. Each node represents an asset and the weighted edge $\left( i, j\right)$ measures the correlation between assets *i* and *j*.

As shown in Fig. 1a (top, right) that covers the window January 2008-December 2008, it is noticeable a higher dependence and a higher volatility of the returns in periods of financial crisis. A prominent volatility is partially observed also in 2020 (see Fig. 1a (bottom, right)) due to the effects of Covid-19 announcements on the financial market.

As described in Sect. 3, we solve a network-based portfolio model where the clustering coefficient is used in the optimal problem to catch the structure of interconnections. Indeed, the intensity of the relations between assets is related to the pairwise correlations, that affect the value of the clustering coefficient and, therefore, the optimal solution.

We report in Fig. 1b the optimal solutions of the sample network-based GMV problem, i.e. NB-sample GMV, for the same windows *w* considered in Fig. 1a. In this network representation, the size of bullets is instead proportional to the allocated weight. We observe that the initial endowment is invested in a limited number of assets. As expected, the approach tends to allocate weights on assets characterized by a low volatility and with a preference on assets that are negatively correlated to the rest of the network.

**Fig. 2:**
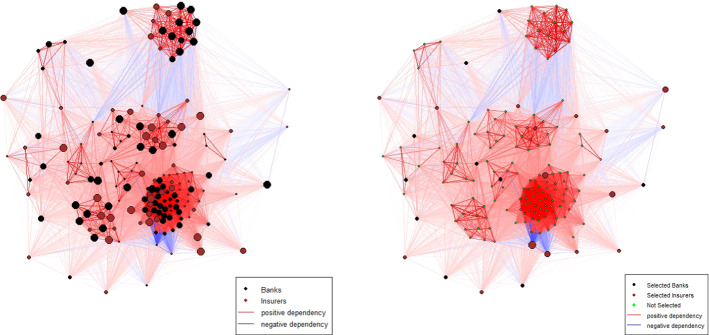
On the left, Pearson Correlation Network computed by using returns of Banks and Insurers dataset (based on sample estimation) referred to the last window, from the beginning of December 2015 to the end of November 2017. Bullets size is proportional to the standard deviation of each firm. Edges opacity is proportional to edges weights (i.e. intensity of correlations). On the right, the optimal network-based sample GMV portfolio (NB-sample GMV) referred to the same period. In this figure, the bullets size is proportional to the allocated weight. Edges opacity is proportional to edges weights

As mentioned before, we will focus in this section on the NKY dataset, but similar results have been obtained for the other datasets. To provide an example, we depict in Fig. 2 the sample correlation network obtained in the window that covers the period December 2015-November 2017 for the Bank and Insurers dataset. In this case, the initial endowment is invested in only 26 firms, specifically 10 banks and 16 insurance companies. However, approximatively 94% of the total amount is invested in insurers that are characterized on average in this time period by both a lower volatility and a lower clustering coefficient.

It is worth noting the case of two insurers, Nationwide Mutual Insurance Company and One America, which are characterized by the lowest standard deviations and a high proportion of negative pairwise correlations (for instance, approximatively 90% of correlations between Nationwide Mutual and other firms is lower than zero). As expected, the optimal portfolio allocates a high proportion of the initial endowment in these two firms (54% and 17% respectively).

In the following we consider the out-of-sample performances of all the models under investigation for the NKY dataset. We analyse the Smart Beta and the MV optimal portfolios obtained using standard and network-based approaches for sample, shrinkage and the weighted $L^2$ depth estimators.

**Fig. 3:**
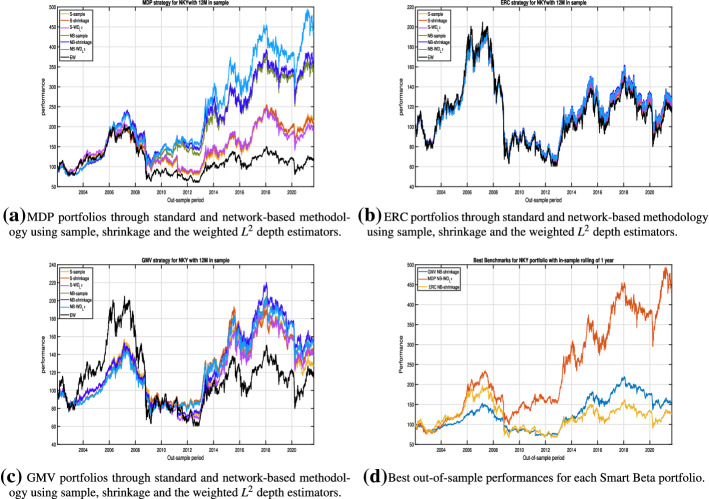
Out-of-sample performances for NKY dataset with a rolling window of 1 year in-sample and 1 month out-sample. In **a**–**c**, we display the out-of-sample performances of EW, S-sample, S-Shrinkage, S-$WD_{L^2}$, NB-sample, NB-Shrinkage and NB-$WD_{L^2}$ of MDP, ERC and GMV models, respectively. In **d** The best out-of-sample performances for each Smart Beta portfolio (MDP, ERC and GMV) are reported

In Fig. 3 the out-of-sample performances of the Smart Beta models under analysis are reported.

We observe that in all cases the EW strategy has the worst performance. Concerning the other strategies, focusing on MDP portfolios (see Fig. 3a) we have a remarkable prevalence of network-based approaches. The improved estimators lead to best performing models over time, with a prevalence of the network-based model (NB-$WD_{L^2}$). However, it should be pointed out that in all the dataset analysed strategies based on shrinkage and $WD_{L^2}$ estimators tend to better perform during and immediately after the sovereign debt crisis.

Among the ERC portfolios (see Fig. 3b), the six alternative strategies show a similar pattern, with a slight preponderance of the NB-shrinkage and NB-$WD_{L^2}$ approaches. Even for the GMV portfolio (see Fig. 3c), the six alternative strategies show a similar pattern, with a slight preponderance of the NB-shrinkage approach.

Figure 3d collects the best out-of-sample performances for each *risk based* analysed portfolio (i.e. MDP, ERC and GMV). What emerges is that the network-based approaches outperform classical strategies and the highest out-of-sample performance at the end of the period is assured by the NB-$WD_{L^2}$ MDP portfolio.

Concluding, from the analysis of the performances of Smart Based portfolios with the alternative approaches, the network-based models almost always lead to higher out-of-sample performance compared to the corresponding classical ones. This result is confirmed also by the analysis carried on the other datasets, for all the estimators of the covariance matrix and for all the rolling windows strategies considered.

However, the simple inspection of the Fig. 3 is not enough in identifying the best portfolio selection strategy. To this end, in order to complete the analysis we report in Table 2 the four moments of the out-of-sample returns’ distributions and values of alternative performance measures (namely, *SR*, *IR*(*EW*)^9^ and *OR*). As well-known, these performance measures consider different characteristics of the portfolios and they could lead to different rankings between the models. However, by the inspection of Table 2, we can provide additional insights.

**Table 2:** Out-of-sample statistics for the *risk-based* approaches in case of NKY portfolio with a buy and hold strategy of 12 months in-sample and 1 month out-of-sample

	S-sample	S-shrinkage	S-$WD_{L^2}$	NB-sample	NB-shrinkage	NB-$WD_{L^2}$	EW
ERC							
$\mu ^{\star }$	0.00012	0.00013	0.00013	0.00014	**0.00015**	0.00014	0.00014
$\sigma ^{\star }$	0.013	0.013	0.013	0.012	0.012	**0.012**	0.014
$skew^{\star }$	$-$ 0.513	$-$ 0.497	$-$ 0.520	$-$ 0.488	$-$ 0.488	$-$ 0.481	$-$ **0.436**
$kurt^{\star }$	12.289	12.068	12.433	11.967	11.967	11.975	**10.949**
$SR^{\star }$	0.009	0.010	0.010	0.010	**0.012**	0.010	0.010
$IR^{\star }(EW)$	$-$ 0.003	$-$ 0.007	$-$ 0.004	0.001	**0.001**	0.000	
$OR^{\star }$	1.028	1.030	1.029	1.031	**1.032**	1.031	1.028
MDP							
$\mu ^{\star }$	0.00022	0.00022	0.00022	0.00032	0.00033	**0.00037**	0.00014
$\sigma ^{\star }$	0.012	0.011	0.012	0.011	0.011	**0.011**	0.014
$skew^{\star }$	$-$ 0.787	$-$ 0.787	$-$ 0.875	$-$ 0.655	$-$ 0.552	$-$ 0.591	$-$ **0.436**
$kurt^{\star }$	12.612	12.918	13.772	11.588	11.355	11.334	**10.949**
$SR^{\star }$	0.018	0.019	0.018	0.026	0.027	**0.030**	0.010
$IR^{\star }(EW)$	0.011	0.014	0.016	0.020	0.023	**0.025**	
$OR^{\star }$	1.055	1.057	1.056	1.080	1.081	**1.090**	1.028
GMV							
$\mu ^{\star }$	0.00012	0.00011	0.00011	0.00013	0.00013	0.00012	**0.00014**
$\sigma ^{\star }$	0.009	0.009	0.010	0.009	**0.008**	**0.008**	0.014
$skew^{\star }$	$-$ 0.564	$-$ 0.477	$-$ 0.576	$-$ 0.404	$-$ 0.404	$-$ **0.325**	$-$ 0.436
$kurt^{\star }$	18.439	18.579	18.769	15.801	15.766	16.428	**10.949**
$SR^{\star }$	0.013	0.012	0.012	**0.016**	**0.016**	0.015	0.010
$IR^{\star }(EW)$	$-$ 0.001	$-$ 0.005	$-$ 0.003	0.000	**0.000**	$-$ 0.002	
$OR^{\star }$	1.041	1.030	1.036	**1.042**	**1.043**	1.041	1.028

For each strategy (ERC, MDP and GMV), sample, shrinkage and $WD_{L^2}$ estimators are reported for classical and network-based models. The last column also considers results for EW. The best results are reported in bold for each measure. All the statistics are reported on daily bases

First, we observe that for all the strategies, the network-based approaches lead to a lower out-of-sample risk, measured by the standard deviation, regardless of the estimation method of the covariance matrix. Moreover, for each strategy the network-based approach almost always leads to a less relevant negative tail, due to a skewness closer to zero and a lower kurtosis with respect to the corresponding standard approach. These findings are further confirmed by *SR* and *OR* values. In particular, it results that the best portfolio is obtained by one of the *Network Based* approaches. In the majority of the results obtained there is a prevalence of the $NB-WD_{L^2}$ and $NB-shrinkage$. Hence, the results reported in Table 2 make us more confident in believing that using the network theory in building the *Smart Beta* strategies can be a good alternative to the standard approach not only for the easier visualization of the results (as reported in Figs. 1 and 3) but also for the better performances that they may reach in an out-of-sample perspective. The conclusions drawn for NKY portfolio with a monthly stepped one-year rolling window are still valid, in general, also for the other rolling windows and portfolios under analysis.^10^

Let us now analyse the results obtained in case of the Mean-Variance portfolio where different levels of trade-off between risk and return are considered. In particular we report the results for $\lambda$ equal to $0.2, \ 0.4,\ 0.6, \ 0.8$, respectively. A low level of $\lambda$ indicates that the investor gives higher importance to the portfolio return. In particular, $\lambda =0$ indicates that the decision maker is completely ignoring the risk of the portfolio. In this case, the optimal portfolio is usually concentrated only in the asset with the higher return.^11^ On the contrary, high values of $\lambda$ indicate a higher relevance to the risk with respect to the return. The extreme case of $\lambda =1$ corresponds to the GMV portfolio, meaning that the decision maker completely ignores the portfolio return.

**Fig. 4:**
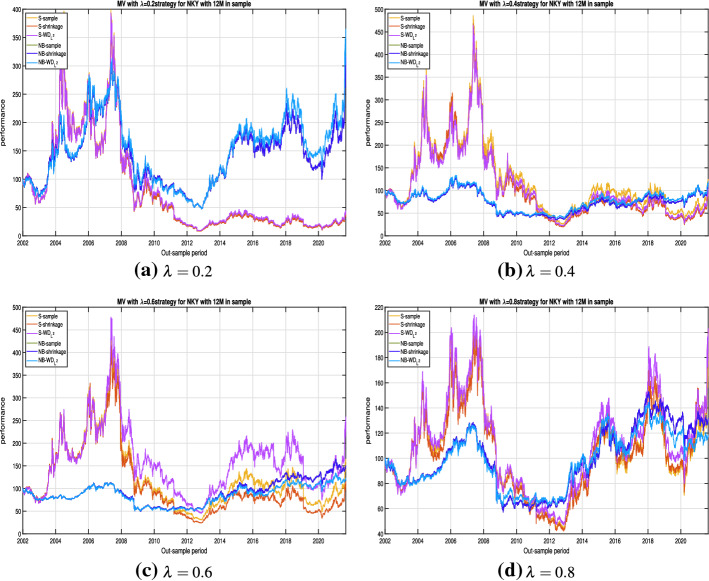
Out-of-sample performances for NKY portfolio with a rolling window of 12 months in-sample and 1 month out-sample. In **a**–**d** we report the out-of-sample performances for $S-sample$, $S-Shrinkage$, $S-WD_{L^2}$, $NB-sample$, $NB-Shrinkage$ and $NB-WD_{L^2}$ strategies according to alternative values of the trade-off parameter (namely, $\lambda =0.2$, $\lambda =0.4$, $\lambda =0.6$ and $\lambda =0.8$ respectively)

To this end, Fig. 4 reports the out-of-sample performances of the NKY dataset, with a buy and hold strategy of 12 months in-sample and one month out-of-sample, in case of the MV model. As previously described in Sect. 2.1, for this strategy ${\varvec{\mu }}$ and ${\varvec{\Sigma }}$ have to be estimated. We estimate ${\varvec{\mu }}$ using the sample approach while ${\varvec{\Sigma }}$ is estimated using the *sample*, the *shrinkage toward constant correlation* and the *weighted-depth* $L^2$ methods. Notice that the matrix ${\varvec{\Sigma }}$ is also used in the network-based approaches to construct the network and to obtain the interconnectedness matrix ${\varvec{C}}$. Figure 4a displays the out-of-sample performances obtained setting $\lambda =0.2$, which means that the investor tends to prefer high potential returns with respect to low levels of uncertainty. Although it is not possible to define a univocal ranking between methods in terms of performance, we observe higher returns at the end of the period with the network-based approaches. Indeed, it is noticeable a fast decrease in the out-of-sample performance of the *Standard approaches* after the crisis of 2008 while the performance of the *Network Based approaches* is much more stable leading at the end of the period at a prevalence of NB models.

Post-crisis effect is also evident for the other values of $\lambda$ (see Fig. 4b–d).

To have a complete view of the effect of the network based strategies on the MV model, we report in Table 3 the first four moments and alternative performance measures.

**Table 3:** Out-of-sample statistics for the MV model, in case of the NKY portfolio with one year rolling estimation window for the mean vector and the covariance matrix and one month for out-of-sample returns

	S-sample	S-shrinkage	S-$WD_{L^2}$	NB-sample	NB-shrinkage	NB-$WD_{L^2}$	S-sample	S-shrinkage	S-$WD_{L^2}$	NB- sample	NB-shrinkage	NB-$WD_{L^2}$
$\lambda =0.2$							$\lambda =0.4$					
$\mu ^{\star }$	0.00032	0.00031	**0.00033**	0.00025	0.00025	0.00028	**0.00043**	0.00038	0.00041	0.00020	0.00024	0.00027
$\sigma ^{\star }$	0.031	0.031	0.031	0.021	**0.021**	**0.021**	0.028	0.028	0.028	0.015	**0.015**	**0.015**
$skew^{\star }$	$-$ 0.744	$-$ 0.744	$-$ 0.737	$-$ 0.269	$-$ **0.264**	$-$ 0.298	$-$ 0.460	$-$ 0.467	$-$ 0.470	$-$ 0.445	$-$ **0.445**	$-$ 0.483
$kurt^{\star }$	13.138	13.090	13.061	7.555	**7.510**	7.632	8.179	8.149	**8.097**	9.002	9.002	9.303
$SR^{\star }$	0.010	0.010	0.011	0.012	0.012	**0.013**	0.016	0.014	0.014	0.014	**0.016**	**0.018**
$IR^{\star }(EW)$	0.007	0.006	0.007	0.018	0.018	**0.019**	0.010	0.011	0.012	0.011	**0.013**	**0.013**
$OR^{\star }$	1.031	1.029	1.032	1.060	1.060	**1.064**	1.046	1.040	1.045	1.039	1.044	**1.046**
$\lambda =0.6$							$\lambda =0.8$					
$\mu ^{\star }$	0.00038	0.00032	**0.00047**	0.00018	0.00021	**0.00024**	0.00028	0.00030	**0.00031**	0.00019	0.00019	**0.00022**
$\sigma ^{\star }$	0.024	0.024	0.024	**0.012**	**0.012**	**0.012**	0.018	0.018	0.019	**0.010**	**0.010**	**0.010**
$skew^{\star }$	$-$ 0.504	$-$ 0.483	$-$ **0.422**	$-$ 0.636	$-$ 0.636	$-$ 0.584	$-$ 0.514	$-$ 0.485	$-$ 0.484	$-$ 0.532	$-$ 0.532	$-$ **0.431**
$kurt^{\star }$	8.329	8.311	**7.466**	12.288	12.288	12.227	8.691	8.671	**8.282**	13.807	13.807	14.158
$SR^{\star }$	0.016	0.013	**0.020**	0.016	0.018	**0.021**	0.016	0.017	0.017	0.018	0.018	**0.021**
$IR^{\star }(EW)$	0.013	0.009	0.017	**0.018**	0.017	0.016	0.011	0.012	**0.013**	0.012	0.012	**0.013**
$OR^{\star }$	1.047	1.039	1.058	**1.059**	1.044	1.058	1.046	1.049	1.049	1.045	1.045	**1.054**

The best results are reported in bold for each measure. All the statistics are reported on daily bases

The results in Table 3 clearly shows that, for all considered values of $\lambda$, the MV network-based portfolio has a lower volatility than the corresponding standard approach. Moreover, in the majority of the cases the network-based portfolios lead also to higher out-of-sample performances in terms of Sharpe ratio and Omega ratio. These results are in line with those obtained for the *risk-based* approaches, presented in Fig. 3 and Table 2, confirming that applying network tools to portfolio selection models may enhance the portfolio selection process.

The use of network tools to manage the optimal portfolio selection proved to be effective, especially in the case of *risk-based* strategies. Looking at the results of the MV portfolios, in the Supplementary Material we can observe that the network-based portfolios lead to better out-of-sample results for some levels of the trade-off parameters. However, there is not an absolute dominance of these approaches, since in some cases the standard methods behave better for specific values of the trade-off parameter. This behaviour depends on the trade-off parameter value and to the estimator used for the portfolio mean.

## Conclusions

In this work we applied network tools to the most used portfolio models characterized by an objective function depending on the covariance matrix of assets. Following Clemente et al. (2019), we took advantage of the correlations network to capture the interconnectedness between assets, that explicitly enters through the clustering coefficient in the objective function. We extended the approach of Clemente et al. (2019), tested to the GMV model, proposing the application of network theory also for the most used Smart Beta models, as well as the MV model. We estimated the network-correlation structure through the sample (as in Clemente et al. 2019), the shrinkage toward the constant correlation and the weighted depth - $L^2$ approaches.

To test the robustness of our methodology, we performed numerical analyses, based on three large-dimensional portfolios. We implemented both the standard and the network-based models, using sample, shrinkage $WD_{L^2}$ estimators for the covariance matrix, and we compared the out-of-sample performances based on a rolling sample optimization. The results obtained show in most cases the effectiveness of network-based portfolios compared to the standard approaches and to the equally weighted portfolios. Network-based strategies show higher out-of-sample performances and lower out-of-sample volatility, reducing the risk. Results appeared significant especially for Smart Beta strategies, which are based only on the risk measure, that refers to the covariance matrix or the interconnectedness matrix. Results in case of mean-variance portfolio do not provide a univocal ranking of the models. However, the network-based approaches lead to better results in the most cases, although there is a good percentage of cases in which the standard approaches behave better. This behaviour depends obviously on the trade-off parameter value and to the estimator used for the portfolio mean. We believe that better out-of-sample results can be obtained in case the network theory is used for the estimation of not only of the risk measure but also of the performance measure, which is left for future research.

In conclusion, we hope that this empirical analysis will help to shed some light on how network theory can be implemented in portfolio selection problems and encourage portfolio managers in considering and testing the network-based portfolio selection models as an alternative to the standard approaches.

## Supplementary Information

Below is the link to the electronic supplementary material.

Supplementary file 1 (pdf 198 KB)

## Footnotes

1 The term *Smart Beta* is popular to denote any strategy which attempts to take advantage of the benefits of traditional passive investments, adding a source of outperformance in order to beat traditional market capitalization-weighted indices. For more information on the *risk-based* strategies see e.g. Amenc and Goltz (2013) and the references therein.

2 For further information on how is estimated the shrinkage intensity $\kappa$ see Ledoit and Michael (2004).

3 The greatest firms by market capitalization in the banking and insurance sector are considered.

4 All the data have been downloaded from Bloomberg (2012).

5 In the literature different lengths for the in-sample and out-of-sample period has been used, see among others Cesarone et al. (2013) and Consigli et al. (2018).

6 In general, the superscript $\star$ indicates that the statistic is calculated using the out-of-sample time series of returns of the optimal portfolio.

7 As a proxy for the risk-free rate the literature suggests the use of *1 month* or *3 months* maturity U.S. Treasury Bills (see e.g Deguest et al. 2013) or, alternatively, an exogenously given value (for instance, $\mu _f=5\%$ is considered in Brennan 1998). In the empirical part of this paper, for illustration purposes, we set $\mu _f=0 \%$.

8 We point out that *OR* ratio is very sensitive to values of $\epsilon$ which can be different from investor to investor. In the empirical analysis $\epsilon$ is set equal to 0.

9 The *IR*(*EW*) indicates the Information ratio where the reference portfolio is the Equally Weighted one.

10 Detailed results are reported in the Supplementary Material.

11 The optimal portfolio when $\lambda =0$ may not be unique, since more than one asset with the highest return can exist.
