# Supplementary material for: Smart network based portfolios
Source: Ann Oper Res. 2022 Apr 11;316(2):1519–41. doi: 10.1007/s10479-022-04675-7 (PMC8995926; doi:10.1007/s10479-022-04675-7)
Supplement: Supplementary file 1 — (pdf 198 KB) [file 10479_2022_4675_MOESM1_ESM.pdf]

Supplementary material for the paper: "Smart network based portfolios"

Gian Paolo Clemente[♮], Rosanna Grassi[♯], Asmerilda Hitaj[◇*]

[♮] Dipartimento di Discipline Matematiche, Finanza Matematica ed Econometria, Università Cattolica del Sacro Cuore, Milano, Italy

Email: gianpaolo.clemente@unicatt.it

[♯] Dipartimento di Statistica e Metodi Quantitativi, Università degli Studi di Milano-Bicocca, Milano, Italy

Email: rosanna.grassi@unimib.it

[◇] Dipartimento di Economia, Università degli studi dell'Insubria, Varese, Italy

Email: asmerilda.hitaj@uninsubria.it

1 Results for the NKY dataset

We report here the results obtained for the NKY dataset based on two alternative rolling windows that have been tested and that are not present in the main part of the paper, i.e. six months or two years in-sample and one month out-of-sample.

[*]Corresponding author.

1.1 Monthly-stepped six months rolling windows

				ERC			
	S-sample	S-shrinkage	S-WD_{L^2}	NB-sample	NB-shrinkage	NB-WD_{L^2}	EW
$\mu^\star$	0.00008	0.00007	0.00008	0.00008	0.00008	0.00008	**0.00008**
$\sigma^\star$	0.013	0.013	**0.013**	0.013	0.013	0.013	0.014
$skew^\star$	-0.534	-0.513	-0.538	-0.510	-0.510	-0.504	**-0.448**
$kurt^\star$	12.188	11.901	12.222	11.923	11.917	11.886	**10.676**
$SR^\star$	0.006	0.005	0.006	0.006	0.006	0.006	0.006
$IR^\star(EW)$	-0.003	-0.007	-0.002	**-0.001**	-0.002	**-0.001**	
$OR^\star$	1.017	1.015	1.018	**1.018**	1.017	**1.018**	1.017
				MDP			
	S-sample	S-shrinkage	S-WD_{L^2}	NB-sample	NB-shrinkage	NB-WD_{L^2}	EW
$\mu^\star$	0.00017	0.00018	0.00012	0.00027	0.00022	**0.00028**	0.00008
$\sigma^\star$	0.012	**0.012**	0.012	0.012	0.012	0.013	0.014
$skew^\star$	-0.759	-0.762	-0.757	-0.579	-0.578	-0.544	**-0.448**
$kurt^\star$	13.703	13.966	13.503	11.708	11.694	10.802	**10.676**
$SR^\star$	0.014	0.016	0.010	**0.022**	0.018	**0.022**	0.006
$IR^\star(EW)$	0.012	0.015	0.005	0.020	0.015	**0.022**	
$OR^\star$	1.043	1.048	1.030	1.066	1.053	**1.068**	1.017
				GMV			
	S-sample	S-shrinkage	S-WD_{L^2}	NB-sample	NB-shrinkage	NB-WD_{L^2}	EW
$\mu^\star$	0.00014	0.00013	0.00014	0.00014	0.00014	**0.00015**	0.00008
$\sigma^\star$	0.009	**0.009**	0.010	0.010	0.010	0.010	0.014
$skew^\star$	-0.577	-0.395	-0.593	-0.346	-0.346	**-0.241**	-0.448
$kurt^\star$	17.562	17.774	19.199	16.424	16.443	15.800	**10.676**
$SR^\star$	0.015	0.014	0.015	0.015	0.014	**0.016**	0.006
$IR^\star(EW)$	0.006	0.006	**0.008**	0.006	0.007	0.008	
$OR^\star$	1.045	1.045	1.046	1.046	1.047	**1.049**	1.017

Table 1: Out-of-sample statistics for the *risk-based* approaches in case of NKY dataset with a buy and hold strategy of 6 months in-sample and 1 month out-of-sample. For each strategy (ERC, MDP and GMV), sample, shrinkage and WD_{L^2} estimators are reported for classical and network-based models. The last column also considers results for EW. The best results are displayed in bold for each measure. All the statistics are reported on daily bases.

	$\lambda = 0.2$						$\lambda = 0.4$					
	S-sample	S-shrinkage	S-WD_{L^2}	NB-sample	NB-shrinkage	NB-WD_{L^2}	S-sample	S-shrinkage	S-WD_{L^2}	NB- sample	NB-shrinkage	NB-WD_{L^2}
$\mu^\star$	0.00009	0.00008	0.00008	0.00020	0.00020	**0.00022**	0.00012	0.00016	0.00016	**0.00021**	0.00020	0.00020
$\sigma^\star$	0.032	0.032	0.032	0.023	0.023	**0.023**	0.030	0.030	0.030	**0.017**	0.017	0.017
$skew^\star$	-0.585	-0.579	-0.580	-0.378	-0.378	**-0.360**	-0.682	-0.684	-0.677	**-0.524**	-0.525	-0.542
$kurt^\star$	12.688	12.627	12.616	7.926	**7.920**	7.922	14.070	14.119	14.006	9.125	**9.121**	9.308
$SR^\star$	0.003	0.002	0.003	0.009	0.009	**0.010**	0.004	0.005	0.005	**0.012**	0.012	0.012
$IR^\star(EW)$	0.000	0.000	0.000	0.006	0.006	**0.007**	0.002	0.003	0.003	**0.010**	0.009	0.009
$OR^\star$	1.009	1.007	1.008	1.026	1.026	**1.028**	1.012	1.016	1.016	**1.037**	1.035	1.035
	$\lambda = 0.6$						$\lambda = 0.8$					
	S-sample	S-shrinkage	S-WD_{L^2}	NB-sample	NB-shrinkage	NB-WD_{L^2}	S-sample	S-shrinkage	S-WD_{L^2}	NB- sample	NB-shrinkage	NB-WD_{L^2}
$\mu^\star$	**0.00029**	0.00029	0.00028	0.00017	0.00016	0.00018	**0.00027**	0.00024	0.00023	0.00015	0.00014	0.00015
$\sigma^\star$	0.026	0.026	0.026	0.013	0.013	**0.013**	0.020	0.020	0.020	0.010	0.010	**0.010**
$skew^\star$	**-0.334**	-0.823	-0.774	-0.730	-0.732	-0.731	**-0.511**	-0.518	-0.517	-0.707	-0.709	-0.552
$kurt^\star$	**8.302**	15.143	14.475	12.001	12.027	12.150	8.340	8.195	**8.091**	16.683	16.734	15.142
$SR^\star$	0.011	0.011	0.011	0.013	0.012	**0.014**	0.014	0.012	0.011	**0.014**	0.014	0.014
$IR^\star(EW)$	0.009	0.009	0.009	0.011	0.012	**0.013**	**0.012**	0.010	0.009	0.007	0.006	0.006
$OR^\star$	1.033	1.032	1.032	**1.038**	1.036	**1.038**	1.040	1.034	1.032	**1.043**	1.041	1.042

Table 2: Out-of-sample statistics for the MV model, in case of the NKY dataset with 6 months rolling estimation window for the mean vector and the covariance matrix and one month for out-of-sample returns. The best results are displayed in bold for each measure. All the statistics are reported on daily bases.

1.2 Monthly-stepped two years rolling windows

				ERC			
	S-sample	S-shrinkage	S-WD_{L^2}	NB-sample	NB-shrinkage	NB-WD_{L^2}	EW
$\mu^\star$	0.00016	0.00016	0.00016	0.00019	**0.00020**	0.00018	0.00018
$\sigma^\star$	0.013	0.013	0.013	0.013	**0.013**	0.013	0.014
$skew^\star$	-0.540	-0.529	-0.543	-0.532	-0.532	-0.518	**-0.470**
$kurt^\star$	12.625	12.496	12.806	12.496	12.496	12.533	**11.472**
$SR^\star$	0.012	0.012	0.013	0.014	**0.015**	**0.014**	0.013
$IR^\star(EW)$	-0.011	-0.011	-0.009	0.003	**0.004**	0.000	
$OR^\star$	1.037	1.037	1.038	1.042	**1.043**	1.041	1.038
				MDP			
	S-sample	S-shrinkage	S-WD_{L^2}	NB-sample	NB-shrinkage	NB-WD_{L^2}	EW
$\mu^\star$	0.00024	0.00021	0.00022	0.00044	0.00041	**0.00046**	0.00018
$\sigma^\star$	0.012	**0.012**	0.012	0.013	0.013	0.013	0.014
$skew^\star$	-0.713	-0.770	-0.798	-0.670	-0.674	-0.569	**-0.470**
$kurt^\star$	11.779	12.645	12.699	**11.465**	11.625	11.548	11.472
$SR^\star$	0.021	0.018	0.018	0.034	0.032	**0.036**	0.013
$IR^\star(EW)$	0.009	0.004	0.006	0.027	0.028	**0.030**	
$OR^\star$	1.063	1.053	1.056	1.105	1.103	**1.111**	1.038
				GMV			
	S-sample	S-shrinkage	S-WD_{L^2}	NB-sample	NB-shrinkage	NB-WD_{L^2}	EW
$\mu^\star$	0.00008	0.00005	0.00007	0.00018	**0.00019**	0.00015	0.00018
$\sigma^\star$	**0.010**	0.010	0.010	0.010	**0.010**	0.010	0.014
$skew^\star$	-0.898	-0.857	-0.872	-0.691	-0.691	-0.540	**-0.470**
$kurt^\star$	22.293	22.403	21.870	16.791	16.791	16.389	**11.472**
$SR^\star$	0.008	0.005	0.007	0.017	**0.019**	0.014	0.013
$IR^\star(EW)$	-0.011	-0.014	-0.013	0.000	**0.000**	-0.004	
$OR^\star$	1.025	1.016	1.022	1.052	**1.053**	1.049	1.038

Table 3: Out-of-sample statistics for the *risk-based* approaches in case of NKY dataset with a buy and hold strategy of 2 years in-sample and 1 month out-of-sample. For each strategy (ERC, MDP and GMV), sample, shrinkage and WD_{L^2} estimators are reported for classical and network-based models. The last column also considers results for EW. The best results are displayed in bold for each measure. All the statistics are reported on daily bases.

	$\lambda = 0.2$						$\lambda = 0.4$					
	S-sample	S-shrinkage	S-WD_{L^2}	NB-sample	NB-shrinkage	NB-WD_{L^2}	S-sample	S-shrinkage	S-WD_{L^2}	NB- sample	NB-shrinkage	NB-WD_{L^2}
$\mu^\star$	0.00041	**0.00042**	0.00040	0.00033	0.00033	0.00032	0.00037	**0.00038**	0.00038	0.00022	0.00022	0.00018
$\sigma^\star$	0.028	0.028	0.029	0.018	**0.018**	**0.018**	0.025	0.025	0.025	0.014	0.014	**0.013**
$skew^\star$	-0.177	**-0.170**	-0.174	-0.544	-0.544	-0.551	-0.365	-0.365	**-0.356**	-0.574	-0.574	-0.590
$kurt^\star$	9.394	9.417	9.345	8.710	**8.700**	8.801	8.354	8.379	**8.285**	10.155	10.155	10.625
$SR^\star$	0.014	0.015	0.014	0.018	**0.018**	0.018	0.015	0.015	0.015	0.016	**0.016**	0.014
$IR^\star(EW)$	0.009	0.010	0.009	0.010	**0.010**	0.010	0.009	**0.010**	0.009	0.004	0.004	0.000
$OR^\star$	1.043	1.044	1.042	1.053	**1.053**	1.052	1.044	1.045	1.044	**1.049**	**1.049**	1.041
	$\lambda = 0.6$						$\lambda = 0.8$					
	S-sample	S-shrinkage	S-WD_{L^2}	NB-sample	NB-shrinkage	NB-WD_{L^2}	S-sample	S-shrinkage	S-WD_{L^2}	NB- sample	NB-shrinkage	NB-WD_{L^2}
$\mu^\star$	0.00038	**0.00039**	0.00036	0.00024	0.00026	0.00025	0.00020	**0.00024**	0.00023	0.00016	0.00016	0.00013
$\sigma^\star$	0.021	0.021	0.021	0.012	0.012	**0.011**	0.016	0.016	0.017	0.011	0.011	**0.011**
$skew^\star$	-0.452	-0.457	**-0.451**	-0.552	-0.552	-0.559	-0.625	-0.611	-0.618	-0.633	-0.633	**-0.519**
$kurt^\star$	8.415	8.471	**8.155**	12.912	12.912	13.745	8.842	8.899	**8.828**	15.398	15.398	15.309
$SR^\star$	0.018	0.019	0.017	0.021	**0.022**	**0.022**	0.013	0.015	0.014	0.015	**0.015**	0.013
$IR^\star(EW)$	0.012	0.012	0.011	0.011	**0.013**	0.009	0.002	**0.005**	0.004	-0.002	-0.002	-0.005
$OR^\star$	1.053	1.055	1.049	1.054	**1.057**	1.050	1.037	1.043	1.041	1.047	**1.047**	1.039

Table 4: Out-of-sample statistics for the MV model, in case of the NKY dataset with 2 years rolling estimation window for the mean vector and the covariance matrix and one month for out-of-sample returns. The best results are displayed in bold for each measure. All the statistics are reported on daily bases.

2 Results for the Banks & Insurers dataset

We report here the results obtained for the Banks and Insurers dataset based on the three alternative rolling windows that have been tested, i.e. six months, one year or two years in-sample and one month out-of-sample.

2.1 Monthly-stepped six months rolling windows

	S-sample	S-shrinkage	S-WD_{L^2}	NB-sample	NB-shrinkage	NB-WD_{L^2}	EW
**ERC**							
$\mu^\star$	0.00006	0.00006	0.00006	0.00005	**0.00007**	0.00007	0.00003
$\sigma^\star$	0.009	0.009	0.009	0.008	**0.007**	0.008	0.012
$skew^\star$	-0.479	-0.444	-0.459	-0.410	-0.410	-0.440	**-0.340**
$kurt^\star$	14.065	13.683	14.460	14.589	14.592	15.380	**12.977**
$SR^\star$	0.007	0.007	0.006	0.007	**0.010**	0.008	0.003
$IR^\star(EW)$	0.008	0.009	0.008	0.009	**0.011**	0.003	
$OR^\star$	1.022	1.022	1.020	**1.023**	**1.023**	1.022	1.009
**MDP**							
	S-sample	S-shrinkage	S-WD_{L^2}	NB-sample	NB-shrinkage	NB-WD_{L^2}	EW
$\mu^\star$	0.00012	0.00020	0.00007	0.00017	**0.00020**	0.00016	0.00003
$\sigma^\star$	0.011	0.007	0.007	0.007	**0.007**	0.010	0.012
$skew^\star$	0.035	1.141	-0.960	**4.237**	1.156	0.802	-0.340
$kurt^\star$	112.707	29.939	24.439	94.046	44.819	59.324	**12.977**
$SR^\star$	0.011	0.029	0.009	0.023	**0.031**	0.017	0.003
$IR^\star(EW)$	0.007	0.018	0.004	0.012	**0.021**	0.010	
$OR^\star$	1.050	1.108	1.031	1.084	**1.111**	1.096	1.089
**GMV**							
	S-sample	S-shrinkage	S-WD_{L^2}	NB-sample	NB-shrinkage	NB-WD_{L^2}	EW
$\mu^\star$	0.00004	0.00004	0.00004	0.00004	**0.00005**	0.00005	0.00003
$\sigma^\star$	0.002	**0.002**	0.002	0.002	0.002	0.002	0.012
$skew^\star$	-0.832	-0.720	-0.835	-0.568	-0.568	-0.680	**-0.340**
$kurt^\star$	13.890	16.536	16.207	16.377	16.377	18.776	**12.977**
$SR^\star$	0.020	0.022	0.016	0.020	**0.023**	0.022	0.003
$IR^\star(EW)$	0.001	**0.001**	0.000	-0.002	-0.002	-0.002	
$OR^\star$	1.061	1.054	1.050	1.063	**1.064**	1.054	1.009

Table 5: Out-of-sample statistics for the *risk-based* approaches in case of Banks and Insurers dataset with a buy and hold strategy of 6 months in-sample and 1 month out-of-sample. For each strategy (ERC, MDP and GMV), sample, shrinkage and WD_{L^2} estimators are reported for classical and network-based models. The last column also considers results for EW. The best results are displayed in bold for each measure. All the statistics are reported on daily bases.

	$\lambda = 0.2$						$\lambda = 0.4$					
	S-sample	S-shrinkage	S-WD_{L^2}	NB-sample	NB-shrinkage	NB-WD_{L^2}	S-sample	S-shrinkage	S-WD_{L^2}	NB- sample	NB-shrinkage	NB-WD_{L^2}
$\mu^\star$	-0.00001	-0.00003	-0.00058	0.00004	0.00004	**0.00011**	0.00011	**0.00012**	-0.00010	0.00002	0.00002	0.00009
$\sigma^\star$	0.028	0.028	0.048	0.021	0.021	**0.020**	0.025	0.026	0.029	0.014	0.014	**0.012**
$skew^\star$	-0.078	-0.076	-10.077	-0.196	-0.196	**-0.041**	**-0.065**	-0.065	-2.563	-0.361	-0.361	-0.319
$kurt^\star$	**9.993**	10.119	386.994	14.192	14.192	14.004	9.391	9.388	66.107	9.535	9.535	**9.041**
$SR^\star$	0.000	-0.001	-0.012	0.002	0.002	**0.006**	0.004	0.005	-0.003	0.001	0.001	**0.007**
$IR^\star(EW)$	-0.002	-0.002	-0.013	0.000	0.000	**0.004**	0.003	0.003	-0.005	-0.001	-0.001	**0.004**
$OR^\star$	0.999	0.997	0.945	1.005	1.005	**1.017**	1.012	1.014	0.989	1.004	1.004	**1.021**
	$\lambda = 0.6$						$\lambda = 0.8$					
	S-sample	S-shrinkage	S-WD_{L^2}	NB-sample	NB-shrinkage	NB-WD_{L^2}	S-sample	S-shrinkage	S-WD_{L^2}	NB- sample	NB-shrinkage	NB-WD_{L^2}
$\mu^\star$	**0.00038**	0.00036	0.00027	0.00008	0.00008	0.00008	0.00025	**0.00027**	0.00022	0.00006	0.00006	0.00005
$\sigma^\star$	0.022	0.022	0.023	0.009	0.009	**0.007**	0.016	0.016	0.017	0.005	0.005	**0.004**
$skew^\star$	**0.007**	-0.013	-0.584	-0.607	-0.607	-0.105	**0.061**	0.050	-0.046	-0.131	-0.131	-0.054
$kurt^\star$	**11.271**	11.377	17.757	14.157	14.157	12.545	**9.899**	9.922	10.075	20.624	20.624	18.449
$SR^\star$	**0.017**	0.016	0.012	0.008	0.008	0.010	**0.016**	0.016	0.013	0.012	0.012	0.014
$IR^\star(EW)$	**0.016**	0.015	0.011	0.004	0.004	0.004	0.014	**0.015**	0.011	0.003	0.003	0.002
$OR^\star$	**1.052**	1.048	1.035	1.027	1.027	1.033	1.046	**1.048**	1.038	1.041	1.041	1.045

Table 6: Out-of-sample statistics for the MV model, in case of the Banks and Insurers dataset with 6 months rolling estimation window for the mean vector and the covariance matrix and one month for out-of-sample returns. The best results are displayed in bold for each measure. All the statistics are reported on daily bases.

2.2 Monthly-stepped 1 year rolling windows

ERC							
	S-sample	S-shrinkage	S-WD_{L^2}	NB-sample	NB-shrinkage	NB-WD_{L^2}	EW
$\mu^\star$	0.00009	0.00007	0.00009	0.00007	0.00007	**0.00010**	0.00005
$\sigma^\star$	0.009	0.009	0.009	**0.009**	0.009	0.009	0.012
$skew^\star$	-0.458	-0.487	-0.458	-0.368	-0.376	-0.411	**-0.324**
$kurt^\star$	14.477	14.448	14.790	14.519	14.554	14.414	**13.126**
$SR^\star$	0.009	0.008	0.010	0.008	0.008	**0.011**	0.005
$IR^\star(EW)$	0.011	0.006	**0.013**	0.005	0.004	0.001	
$OR^\star$	1.029	1.024	**1.031**	1.025	1.025	1.019	1.014
MDP							
	S-sample	S-shrinkage	S-WD_{L^2}	NB-sample	NB-shrinkage	NB-WD_{L^2}	EW
$\mu^\star$	**0.00011**	0.00005	0.00008	0.00004	-0.00001	0.00008	0.00005
$\sigma^\star$	0.008	**0.006**	0.007	0.007	0.007	0.010	0.012
$skew^\star$	-10.095	-0.823	-2.883	-2.288	-1.792	**0.968**	-0.324
$kurt^\star$	417.291	25.828	97.861	39.496	30.759	140.592	**13.126**
$SR^\star$	**0.013**	0.008	0.012	0.005	-0.001	0.008	0.005
$IR^\star(EW)$	**0.006**	-0.001	0.003	-0.002	-0.006	0.002	
$OR^\star$	**1.057**	1.026	1.043	1.019	0.995	1.037	1.014
GMV							
	S-sample	S-shrinkage	S-WD_{L^2}	NB-sample	NB-shrinkage	NB-WD_{L^2}	EW
$\mu^\star$	0.00003	0.00002	0.00002	-0.00002	-0.00002	-0.00003	**0.00005**
$\sigma^\star$	0.002	0.002	0.002	0.002	**0.002**	0.002	0.012
$skew^\star$	-1.236	-1.231	-1.257	-1.233	-1.233	-1.474	**-0.324**
$kurt^\star$	19.485	20.920	23.686	19.837	19.837	24.211	**13.126**
$SR^\star$	**0.013**	0.008	0.007	-0.011	-0.011	-0.012	0.005
$IR^\star(EW)$	**-0.002**	-0.003	-0.003	-0.006	-0.006	-0.006	
$OR^\star$	**1.038**	1.023	1.023	0.968	0.968	0.966	1.014

Table 7: Out-of-sample statistics for the *risk-based* approaches in case of Banks and Insurers dataset with a buy and hold strategy of 1 year in-sample and 1 month out-of-sample. For each strategy (ERC, MDP and GMV), sample, shrinkage and WD_{L^2} estimators are reported for classical and network-based models. The last column also considers results for EW. The best results are reported in bold for each measure. All the statistics are reported on daily bases.

	$\lambda = 0.2$						$\lambda = 0.4$					
	S-sample	S-shrinkage	S-WD_{L^2}	NB-sample	NB-shrinkage	NB-WD_{L^2}	S-sample	S-shrinkage	S-WD_{L^2}	NB- sample	NB-shrinkage	NB-WD_{L^2}
$\mu^\star$	-0.00020	-0.00020	-0.00052	-0.00006	-0.00006	**-0.00002**	-0.00014	-0.00015	-0.00026	0.00005	0.00005	**0.00006**
$\sigma^\star$	0.026	0.026	0.032	0.017	0.017	**0.016**	0.022	0.022	0.024	0.011	0.011	**0.009**
$skew^\star$	0.139	**0.147**	-3.122	-0.382	-0.382	-0.392	0.111	**0.118**	-0.342	-0.507	-0.507	-0.537
$kurt^\star$	7.786	7.836	90.025	8.835	8.835	**7.783**	**7.809**	7.822	12.559	11.975	11.975	9.213
$SR^\star$	-0.008	-0.008	-0.017	-0.004	-0.004	**-0.001**	-0.006	-0.007	-0.011	0.004	0.004	**0.007**
$IR^\star(EW)$	-0.010	-0.010	-0.019	-0.007	-0.007	**-0.005**	-0.009	-0.009	-0.014	0.000	0.000	**0.001**
$OR^\star$	0.977	0.977	0.945	0.990	0.990	**0.997**	0.982	0.981	0.968	1.014	1.014	**1.020**
	$\lambda = 0.6$						$\lambda = 0.8$					
	S-sample	S-shrinkage	S-WD_{L^2}	NB-sample	NB-shrinkage	NB-WD_{L^2}	S-sample	S-shrinkage	S-WD_{L^2}	NB- sample	NB-shrinkage	NB-WD_{L^2}
$\mu^\star$	0.00001	0.00004	-0.00002	0.00007	**0.00007**	0.00005	0.00021	0.00023	0.00017	0.00005	0.00005	**0.00002**
$\sigma^\star$	0.018	0.019	0.019	0.007	0.007	**0.005**	0.014	0.014	0.014	0.003	0.003	**0.002**
$skew^\star$	-0.020	**0.048**	-0.016	-1.014	-1.014	-0.809	-0.099	**-0.089**	-0.146	-0.890	-0.890	-0.577
$kurt^\star$	7.189	7.214	**7.165**	25.991	25.991	10.757	7.708	7.671	**7.518**	20.696	20.696	8.338
$SR^\star$	0.000	0.002	-0.001	0.010	0.010	**0.011**	0.015	**0.017**	0.013	0.014	0.014	0.009
$IR^\star(EW)$	-0.003	-0.001	-0.004	0.001	**0.001**	0.000	0.011	**0.013**	0.009	0.000	0.000	-0.003
$OR^\star$	1.001	1.005	0.997	1.033	**1.033**	1.033	1.044	**1.049**	1.036	1.046	1.046	1.026

Table 8: Out-of-sample statistics for the MV model, in case of the Banks and Insurers dataset with 1 year rolling estimation window for the mean vector and the covariance matrix and one month for out-of-sample returns. The best results are reported in bold for each measure. All the statistics are reported on daily bases.

2.3 Monthly-stepped 2 year rolling windows

	S-sample	S-shrinkage	S-WD_{L^2}	NB-sample	NB-shrinkage	NB-WD_{L^2}	EW
**ERC**							
$\mu^\star$	0.00014	0.00012	0.00015	0.00012	0.00012	**0.00015**	0.00013
$\sigma^\star$	0.00949	0.00949	0.00967	0.00851	**0.00851**	0.00900	0.01171
$skew^\star$	-0.50079	-0.48999	-0.48388	-0.44457	-0.44413	-0.45184	**-0.41859**
$kurt^\star$	15.06752	15.41191	15.43330	15.27364	15.31783	15.84202	**14.26451**
$SR^\star$	0.01461	0.01253	0.01517	0.01353	0.01379	**0.01707**	0.01117
$IR^\star(EW)$	0.00270	-0.00413	-0.00411	-0.00436	-0.00378	**0.00598**	
$OR^\star$	1.04688	1.04038	1.04906	1.04349	1.04436	**1.04962**	1.03555
**MDP**							
	S-sample	S-shrinkage	S-WD_{L^2}	NB-sample	NB-shrinkage	NB-WD_{L^2}	EW
$\mu^\star$	0.00011	0.00012	0.00015	0.00015	**0.00016**	0.00007	0.00013
$\sigma^\star$	0.00596	0.00561	**0.00506**	0.00845	0.00530	0.01282	0.01171
$skew^\star$	-0.24611	**0.24515**	-0.21510	0.12096	-0.37623	-2.14657	-0.41859
$kurt^\star$	17.69282	31.91482	**14.79651**	36.37598	29.69041	98.75671	14.26451
$SR^\star$	0.01788	0.02066	0.02936	0.01749	**0.02940**	0.00558	0.01117
$IR^\star(EW)$	-0.00250	-0.00157	-0.00192	0.00145	**0.00148**	-0.00843	
$OR^\star$	1.06087	1.07233	1.09689	1.07272	**1.09851**	1.00296	1.03555
**GMV**							
	S-sample	S-shrinkage	S-WD_{L^2}	NB-sample	NB-shrinkage	NB-WD_{L^2}	EW
$\mu^\star$	-0.00001	-0.00001	-0.00001	0.00004	0.00003	0.00004	**0.00013**
$\sigma^\star$	0.00213	0.00203	0.00216	0.00201	**0.00197**	0.00209	0.01171
$skew^\star$	-0.57362	-0.50355	-0.56526	-0.67672	-0.60155	-0.63263	**-0.41859**
$kurt^\star$	**9.40219**	**9.40229**	10.39083	12.43302	11.39856	14.84028	14.26451
$SR^\star$	-0.00434	-0.00432	-0.00555	**0.02085**	0.01758	0.01975	0.01117
$IR^\star(EW)$	-0.01152	-0.01142	-0.01173	**-0.00796**	-0.00852	-0.00818	
$OR^\star$	0.98771	0.98562	0.98421	**1.06285**	1.05262	1.06106	1.03555

Table 9: Out-of-sample statistics for the *risk-based* approaches in case of Banks and Insurers dataset with a buy and hold strategy of 2 years in-sample and 1 month out-of-sample. For each strategy (ERC, MDP and GMV), sample, shrinkage and WD_{L^2} estimators are reported for classical and network-based models. The last column also considers results for EW. The best results are reported in bold for each measure. All the statistics are reported on daily bases.

	$\lambda = 0.2$						$\lambda = 0.4$					
	S-sample	S-shrinkage	S-WD_{L^2}	NB-sample	NB-shrinkage	NB-WD_{L^2}	S-sample	S-shrinkage	S-WD_{L^2}	NB- sample	NB-shrinkage	NB-WD_{L^2}
$\mu^\star$	-0.00008	-0.00009	-0.00025	-0.00002	-0.00002	**0.00012**	-0.00010	-0.00010	-0.00015	0.00007	**0.00007**	0.00005
$\sigma^\star$	0.025	0.025	0.028	0.015	0.015	**0.013**	0.020	0.020	0.021	0.009	0.009	**0.006**
$skew^\star$	0.257	**0.271**	0.048	-0.951	-0.951	-0.638	**-0.207**	-0.248	-0.497	-0.889	-0.889	-0.505
$kurt^\star$	36.962	37.109	33.973	15.327	15.327	**11.029**	15.566	15.214	18.163	24.483	24.483	**8.830**
$SR^\star$	-0.003	-0.004	-0.009	-0.002	-0.002	**0.009**	-0.005	-0.005	-0.007	0.008	**0.008**	0.009
$IR^\star(EW)$	-0.009	-0.009	-0.014	-0.010	-0.010	**-0.001**	-0.012	-0.012	-0.014		**-0.005**	-0.007
$OR^\star$	0.989	0.988	0.970	0.995	0.995	**1.027**	0.985	0.984	0.979	1.026	1.026	**1.026**
	$\lambda = 0.6$						$\lambda = 0.8$					
	S-sample	S-shrinkage	S-WD_{L^2}	NB-sample	NB-shrinkage	NB-WD_{L^2}	S-sample	S-shrinkage	S-WD_{L^2}	NB- sample	NB-shrinkage	NB-WD_{L^2}
$\mu^\star$	0.00008	**0.00008**	0.00002	0.00005	0.00005	0.00002	**0.00023**	**0.00023**	0.00020	0.00001	0.00001	0.00000
$\sigma^\star$	0.016	0.016	0.017	0.005	0.005	**0.003**	0.012	0.012	0.012	0.003	0.003	**0.002**
$skew^\star$	**-0.360**	-0.382	-0.482	-1.044	-1.044	-0.469	-0.437	-0.437	-0.426	-0.699	-0.699	**-0.313**
$kurt^\star$	11.087	10.934	11.656	40.750	40.750	**7.066**	10.241	10.254	10.057	17.167	17.167	**7.079**
$SR^\star$	0.005	0.005	0.001	0.009	**0.009**	0.008	**0.020**	0.019	0.017	0.005	0.005	0.001
$IR^\star(EW)$	**-0.004**	**-0.004**	-0.007	-0.007	-0.007	-0.009	**0.008**	**0.008**	0.006	-0.010	-0.010	-0.011
$OR^\star$	1.015	1.015	1.004	1.032	**1.032**	1.022	**1.062**	1.061	1.052	1.014	1.014	1.002

Table 10: Out-of-sample statistics for the MV model, in case of the Banks and Insurers dataset with 2 years rolling estimation window for the mean vector and the covariance matrix and one month for out-of-sample returns. The best results are reported in bold for each measure. All the statistics are reported on daily bases.

3 Results for the S & P 100 dataset

We report here the results obtained for the S & P 100 dataset based on the three alternative rolling windows that have been tested, i.e. six months, one year or two years in-sample and one month out-of-sample.

3.1 Monthly-stepped six months rolling windows

	S-sample	S-shrinkage	S-WD_{L^2}	NB-sample	NB-shrinkage	NB-WD_{L^2}	EW
**ERC**							
$\mu^\star$	0.00033	0.00032	0.00033	0.00032	0.00032	0.00033	**0.00034**
$\sigma^\star$	**0.011**	0.011	0.011	0.011	0.011	0.011	0.012
$skew^\star$	-0.421	-0.415	-0.399	-0.419	-0.419	-0.397	**-0.396**
$kurt^\star$	18.296	18.079	18.579	17.485	17.485	17.682	**16.263**
$SR^\star$	0.030	0.029	0.030	0.028	0.028	**0.030**	0.027
$IR^\star(EW)$	-0.003	-0.009	-0.002	-0.012	-0.012	**-0.002**	
$OR^\star$	1.102	1.097	1.102	1.095	1.095	**1.105**	1.091
**MDP**							
	S-sample	S-shrinkage	S-WD_{L^2}	NB-sample	NB-shrinkage	NB-WD_{L^2}	EW
$\mu^\star$	0.00048	0.00047	0.00047	0.00048	0.00048	**0.00049**	0.00034
$\sigma^\star$	0.011	0.011	0.011	0.011	0.010	**0.010**	0.012
$skew^\star$	-0.432	-0.433	-0.398	-0.211	**-0.211**	-0.242	-0.396
$kurt^\star$	16.104	17.068	17.130	13.689	13.699	**13.532**	16.263
$SR^\star$	0.045	0.045	0.044	0.042	0.046	**0.047**	0.027
$IR^\star(EW)$	0.026	0.025	0.024	0.022	0.022	**0.026**	
$OR^\star$	1.150	1.140	1.146	1.141	1.142	**1.150**	1.091
**GMV**							
	S-sample	S-shrinkage	S-WD_{L^2}	NB-sample	NB-shrinkage	NB-WD_{L^2}	EW
$\mu^\star$	0.00025	0.00023	0.00026	0.00021	0.00021	0.00021	**0.00034**
$\sigma^\star$	0.008	0.008	0.008	0.008	**0.008**	0.008	0.012
$skew^\star$	-0.169	-0.108	**0.023**	-0.119	-0.119	0.016	-0.396
$kurt^\star$	21.110	22.797	24.131	19.941	19.941	21.647	**16.263**
$SR^\star$	0.030	0.029	**0.031**	0.024	0.024	0.025	0.027
$IR^\star(EW)$	-0.012	-0.014	**-0.011**	-0.017	-0.017	-0.016	
$OR^\star$	1.098	1.094	**1.103**	1.078	1.078	1.081	1.091

Table 11: Out-of-sample statistics for the *risk-based* approaches in case of S&P dataset with a buy and hold strategy of 6 months in-sample and 1 month out-of-sample. For each strategy (ERC, MDP and GMV), sample, shrinkage and WD_{L^2} estimators are reported for classical and network-based models. The last column also considers results for EW. The best results are reported in bold for each measure. All the statistics are reported on daily bases.

	$\lambda = 0.2$						$\lambda = 0.4$					
	S-sample	S-shrinkage	S-WD_{L^2}	NB-sample	NB-shrinkage	NB-WD_{L^2}	S-sample	S-shrinkage	S-WD_{L^2}	NB- sample	NB-shrinkage	NB-WD_{L^2}
$\mu^\star$	0.00131	**0.00133**	0.00131	0.00051	0.00051	0.00048	**0.00122**	**0.00122**	**0.00122**	0.00031	0.00031	0.00029
$\sigma^\star$	0.030	0.030	0.030	0.014	0.014	**0.014**	0.028	0.028	0.028	0.011	0.011	**0.010**
$skew^\star$	-0.214	**-0.209**	-0.217	-0.298	-0.298	-0.349	-0.114	**-0.105**	-0.111	-0.365	-0.365	-0.370
$kurt^\star$	13.297	13.284	13.303	13.100	13.100	**12.308**	13.139	13.112	**13.074**	15.611	15.611	15.539
$SR^\star$	0.044	**0.044**	0.043	0.036	0.036	0.035	0.043	0.043	**0.043**	0.030	0.030	0.028
$IR^\star(EW)$	0.036	**0.037**	0.036	0.016	0.016	0.014	0.036	0.036	**0.036**	-0.003	-0.003	-0.005
$OR^\star$	1.144	**1.145**	1.143	1.116	1.116	1.111	1.143	1.143	**1.143**	1.096	1.096	1.091

	$\lambda = 0.6$						$\lambda = 0.8$					
	S-sample	S-shrinkage	S-WD_{L^2}	NB-sample	NB-shrinkage	NB-WD_{L^2}	S-sample	S-shrinkage	S-WD_{L^2}	NB- sample	NB-shrinkage	NB-WD_{L^2}
$\mu^\star$	0.00098	0.00098	**0.00100**	0.00022	0.00022	0.00022	0.00074	0.00072	**0.00076**	0.00020	0.00020	0.00021
$\sigma^\star$	0.024	0.024	0.024	0.009	0.009	**0.009**	0.018	0.018	0.018	0.009	0.009	**0.008**
$skew^\star$	**0.044**	0.037	-0.011	-0.298	-0.298	-0.176	0.249	**0.251**	0.229	-0.242	-0.242	-0.071
$kurt^\star$	**14.518**	14.577	14.724	17.137	17.137	18.799	18.059	18.751	**17.764**	19.274	19.274	21.286
$SR^\star$	0.041	0.040	**0.041**	0.024	0.024	0.025	0.041	0.040	**0.042**	0.024	0.024	0.025
$IR^\star(EW)$	0.031	0.031	**0.032**	-0.014	-0.014	-0.015	0.028	0.027	**0.029**	-0.018	-0.018	-0.016
$OR^\star$	1.132	1.131	**1.134**	1.078	1.078	1.080	1.131	1.127	**1.132**	1.075	1.075	1.080

Table 12: Out-of-sample statistics for the MV model, in case of the S&P dataset with 6 months rolling estimation window for the mean vector and the covariance matrix and one month for out-of-sample returns. The best results are reported in bold for each measure. All the statistics are reported on daily bases.

3.2 Monthly-stepped 1 year rolling windows

	S-sample	S-shrinkage	S-WD_{L^2}	NB-sample	NB-shrinkage	NB-WD_{L^2}	EW
**ERC**							
$\mu^\star$	0.00034	0.00033	0.00034	0.00033	0.00033	0.00033	**0.00035**
$\sigma^\star$	0.011	0.011	0.011	0.011	**0.010**	0.010	0.012
$skew^\star$	-0.416	-0.406	-0.400	-0.406	-0.406	-0.381	**-0.377**
$kurt^\star$	18.357	18.185	18.565	17.460	17.460	17.714	**16.511**
$SR^\star$	0.031	0.030	0.031	0.030	**0.033**	0.033	0.028
$IR^\star(EW)$	-0.005	-0.009	**-0.005**	-0.008	-0.008	-0.009	
$OR^\star$	1.102	1.103	1.103	1.100	**1.104**	1.032	1.094
**MDP**							
	S-sample	S-shrinkage	S-WD_{L^2}	NB-sample	NB-shrinkage	NB-WD_{L^2}	EW
$\mu^\star$	0.00051	0.00050	0.00051	0.00052	**0.00054**	0.00052	0.00035
$\sigma^\star$	0.011	**0.011**	0.011	0.012	0.012	**0.011**	0.012
$skew^\star$	-0.454	-0.481	-0.447	-0.312	-0.312	**-0.286**	-0.377
$kurt^\star$	17.284	17.957	18.312	**11.760**	11.759	13.245	16.511
$SR^\star$	0.048	0.047	0.048	0.045	**0.047**	0.046	0.028
$IR^\star(EW)$	0.031	0.031	0.030	0.032	**0.033**	0.032	
$OR^\star$	1.155	1.158	1.156	1.157	**1.159**	1.159	1.094
**GMV**							
	S-sample	S-shrinkage	S-WD_{L^2}	NB-sample	NB-shrinkage	NB-WD_{L^2}	EW
$\mu^\star$	0.00023	0.00022	0.00025	0.00022	0.00022	0.00024	**0.00035**
$\sigma^\star$	0.008	0.008	0.008	0.008	**0.007**	0.008	0.012
$skew^\star$	-0.455	-0.354	-0.429	-0.190	-0.190	**-0.131**	-0.377
$kurt^\star$	28.745	27.962	30.433	22.516	22.516	23.905	**16.511**
$SR^\star$	0.027	0.026	0.030	0.027	**0.032**	0.029	0.028
$IR^\star(EW)$	-0.017	-0.018	-0.014	-0.016	-0.016	**-0.014**	
$OR^\star$	1.090	1.087	1.100	1.085	**1.111**	1.109	1.094

Table 13: Out-of-sample statistics for the *risk-based* approaches in case of S&P dataset with a buy and hold strategy of 1 year in-sample and 1 month out-of-sample. For each strategy (ERC, MDP and GMV), sample, shrinkage and WD_{L^2} estimators are reported for classical and network-based models. The last column also considers results for EW. The best results are displayed in bold for each measure. All the statistics are reported on daily bases.

	$\lambda = 0.2$						$\lambda = 0.4$					
	S-sample	S-shrinkage	S-WD_{L^2}	NB-sample	NB-shrinkage	NB-WD_{L^2}	S-sample	S-shrinkage	S-WD_{L^2}	NB- sample	NB-shrinkage	NB-WD_{L^2}
$\mu^\star$	0.00122	**0.00122**	0.00121	0.00054	0.00054	0.00049	0.00127	**0.00127**	0.00127	0.00032	0.00032	0.00031
$\sigma^\star$	0.029	0.029	0.029	0.013	0.013	**0.013**	0.027	0.027	0.027	0.010	0.010	**0.009**
$skew^\star$	-0.177	**-0.176**	-0.179	-0.358	-0.358	-0.307	0.101	**0.108**	0.098	-0.464	-0.464	-0.351
$kurt^\star$	13.278	**13.253**	13.259	14.170	14.170	13.327	11.718	11.712	**11.677**	17.457	17.457	18.753
$SR^\star$	**0.041**	**0.041**	**0.041**	**0.041**	**0.041**	0.039	0.047	**0.048**	0.047	0.033	0.033	0.033
$IR^\star(EW)$	**0.033**	**0.033**	**0.033**	0.019	0.019	0.015	**0.039**	**0.039**	**0.039**	-0.003	-0.003	-0.004
$OR^\star$	**1.134**	**1.134**	1.133	1.132	1.132	1.123	**1.153**	**1.153**	**1.153**	1.106	1.106	1.107
	$\lambda = 0.6$						$\lambda = 0.8$					
	S-sample	S-shrinkage	S-WD_{L^2}	NB-sample	NB-shrinkage	NB-WD_{L^2}	S-sample	S-shrinkage	S-WD_{L^2}	NB- sample	NB-shrinkage	NB-WD_{L^2}
$\mu^\star$	0.00109	**0.00110**	0.00111	0.00024	0.00024	0.00024	0.00067	0.00066	**0.00071**	0.00022	0.00022	0.00024
$\sigma^\star$	0.023	0.023	0.023	**0.009**	**0.009**	**0.009**	0.016	0.016	0.017	**0.009**	**0.009**	0.008
$skew^\star$	**0.138**	0.131	0.118	-0.324	-0.324	-0.225	0.029	**0.070**	0.005	-0.232	-0.232	-0.164
$kurt^\star$	**12.288**	12.490	12.339	20.579	20.579	22.744	**12.526**	13.370	12.672	21.918	21.918	23.795
$SR^\star$	**0.048**	**0.048**	**0.048**	0.027	0.027	0.028	**0.041**	**0.041**	**0.043**	0.026	0.026	0.028
$IR^\star(EW)$	**0.039**	**0.039**	**0.039**	-0.014	-0.014	-0.014	**0.025**	**0.025**	**0.028**	-0.016	-0.016	-0.014
$OR^\star$	**1.152**	**1.153**	**1.153**	1.088	1.088	1.090	1.126	1.126	**1.132**	1.085	1.085	1.091

Table 14: Out-of-sample statistics for the MV model, in case of the S&P dataset with 1 year rolling estimation window for the mean vector and the covariance matrix and one month for out-of-sample returns. The best results are displayed in bold for each measure. All the statistics are reported on daily bases.

3.3 Monthly-stepped 2 year rolling windows

			ERC				
	S-sample	S-shrinkage	S-WD_{L^2}	NB-sample	NB-shrinkage	NB-WD_{L^2}	EW
$\mu^\star$	0.00038	0.00038	0.00038	0.00038	0.00038	0.00038	**0.00041**
$\sigma^\star$	0.011	0.011	0.011	**0.010**	0.011	0.011	0.012
$skew^\star$	-0.494	-0.487	-0.480	-0.496	-0.496	**-0.466**	**-0.460**
$kurt^\star$	19.995	19.908	20.168	**19.111**	19.111	19.449	**18.241**
$SR^\star$	0.035	0.034	0.035	**0.036**	0.034	0.034	0.033
$IR^\star(EW)$	-0.014	-0.017	-0.015	**-0.013**	-0.015	-0.018	
$OR^\star$	**1.119**	1.118	1.119	**1.126**	1.125	1.121	1.114
			MDP				
	S-sample	S-shrinkage	S-WD_{L^2}	NB-sample	NB-shrinkage	NB-WD_{L^2}	EW
$\mu^\star$	0.00056	0.00055	0.00055	**0.00058**	0.00057	0.00054	0.00041
$\sigma^\star$	0.011	0.011	0.011	**0.011**	0.011	0.011	0.012
$skew^\star$	-0.551	-0.570	-0.530	-0.504	-0.570	-0.537	**-0.460**
$kurt^\star$	18.694	19.308	19.699	15.477	**15.477**	17.520	18.241
$SR^\star$	0.053	0.052	0.051	**0.055**	0.051	0.049	0.033
$IR^\star(EW)$	0.031	0.029	0.029	**0.036**	0.035	0.032	
$OR^\star$	1.179	1.176	1.174	**1.180**	1.177	1.176	1.114
			GMV				
	S-sample	S-shrinkage	S-WD_{L^2}	NB-sample	NB-shrinkage	NB-WD_{L^2}	EW
$\mu^\star$	0.00022	0.00021	0.00025	0.00024	0.00024	**0.00025**	**0.00041**
$\sigma^\star$	0.009	**0.009**	0.009	0.009	0.009	0.009	0.012
$skew^\star$	-0.718	-0.722	-0.686	-0.976	-0.976	-0.973	**-0.460**
$kurt^\star$	35.592	35.307	35.689	27.359	27.359	28.474	**18.241**
$SR^\star$	0.026	0.025	0.029	0.027	0.027	**0.029**	0.033
$IR^\star(EW)$	-0.027	-0.028	-0.023	-0.024	-0.024	**-0.022**	
$OR^\star$	1.088	1.084	1.100	1.109	1.129	**1.130**	1.114

Table 15: Out-of-sample statistics for the *risk-based* approaches in case of S&P dataset with a buy and hold strategy of 2 years in-sample and 1 month out-of-sample. For each strategy (ERC, MDP and GMV), sample, shrinkage and WD_{L^2} estimators are reported for classical and network-based models. The last column also considers results for EW. The best results are displayed in bold for each measure. All the statistics are reported on daily bases.

	$\lambda = 0.2$						$\lambda = 0.4$					
	S-sample	S-shrinkage	S-WD_{L^2}	NB-sample	NB-shrinkage	NB-WD_{L^2}	S-sample	S-shrinkage	S-WD_{L^2}	NB- sample	NB-shrinkage	NB-WD_{L^2}
$\mu^\star$	0.00109	0.00109	**0.00110**	0.00056	0.00056	0.00050	0.00099	0.00099	**0.00101**	0.00037	0.00037	0.00033
$\sigma^\star$	0.028	0.028	0.029	0.012	0.012	**0.011**	0.026	0.026	0.026	**0.009**	**0.009**	**0.009**
$skew^\star$	**-0.165**	-0.168	-0.168	-0.278	-0.278	-0.322	**0.022**	**0.021**	**0.028**	-0.423	-0.423	-0.437
$kurt^\star$	11.727	11.746	**11.711**	12.896	12.896	13.900	**11.755**	**11.769**	**11.671**	19.403	19.403	20.909
$SR^\star$	0.038	0.038	0.038	**0.048**	**0.048**	0.045	0.038	0.038	0.039	**0.039**	**0.039**	0.036
$IR^\star(EW)$	**0.028**	**0.028**	**0.028**	0.018	0.018	0.012	**0.027**	0.026	**0.027**	-0.005	-0.005	-0.011
$OR^\star$	1.121	1.120	1.121	**1.155**	**1.155**	1.143	1.120	1.119	1.121	**1.127**	**1.127**	1.116
	$\lambda = 0.6$						$\lambda = 0.8$					
	S-sample	S-shrinkage	S-WD_{L^2}	NB-sample	NB-shrinkage	NB-WD_{L^2}	S-sample	S-shrinkage	S-WD_{L^2}	NB- sample	NB-shrinkage	NB-WD_{L^2}
$\mu^\star$	0.00090	0.00089	**0.00091**	0.00028	0.00028	0.00027	0.00064	0.00063	**0.00067**	0.00035	0.00035	0.00036
$\sigma^\star$	0.021	0.021	0.021	**0.009**	**0.009**	**0.009**	0.015	0.015	0.015	0.008	0.008	**0.007**
$skew^\star$	0.067	**0.085**	0.048	-0.731	-0.731	-0.760	-0.129	**-0.106**	-0.129	-0.803	-0.803	-0.812
$kurt^\star$	**10.496**	10.734	10.528	24.105	24.105	25.139	10.471	10.617	**10.253**	26.090	26.090	27.158
$SR^\star$	**0.043**	0.042	**0.043**	0.031	0.031	0.030	0.044	0.043	0.045	0.045	0.045	**0.050**
$IR^\star(EW)$	**0.029**	0.028	**0.029**	-0.018	-0.018	-0.019	0.023	0.021	**0.024**	-0.022	-0.022	-0.021
$OR^\star$	1.132	1.131	**1.133**	1.103	1.103	1.100	1.135	1.131	**1.137**	1.111	1.101	1.110

Table 16: Out-of-sample statistics for the MV model, in case of the S&P dataset with 2 years rolling estimation window for the mean vector and the covariance matrix and one month for out-of-sample returns. The best results are displayed in bold for each measure. All the statistics are reported on daily bases.
